# Evolving general cooperation with a Bayesian theory of mind

**DOI:** 10.1073/pnas.2400993122

**Published:** 2025-06-16

**Authors:** Max Kleiman-Weiner, Alejandro Vientós, David G. Rand, Joshua B. Tenenbaum

**Affiliations:** ^a^Department of Brain and Cognitive Sciences, Massachusetts Institute of Technology, Cambridge, MA 02139; ^b^Department of Marketing and International Business, Foster School of Business, University of Washington, Seattle, WA 98195; ^c^Paul G. Allen School of Computer Science & Engineering, University of Washington, Seattle, WA 98195; ^d^Sloan School of Management, Massachusetts Institute of Technology, Cambridge, MA 02139; ^e^Institute for Data, Systems, and Society, Massachusetts Institute of Technology, Cambridge, MA 02139

**Keywords:** theory of mind, cooperation, Bayesian models, evolutionary game theory, cognitive science

## Abstract

Theories of the evolution of cooperation through reciprocity explain how unrelated self-interested individuals can accomplish more together than they can on their own. The most prominent theories of reciprocity, such as tit-for-tat or win-stay-lose-shift, are inflexible automata that lack a theory of mind—the human ability to infer the hidden mental states in others’ minds. Here, we develop a model of reciprocity with a theory of mind, the Bayesian Reciprocator. When making decisions, this model does not simply seek to maximize its own payoff. Instead, it also values the payoffs of others—but only to the extent it believes that those others are also cooperating in the same way. To compute its beliefs about others, the Bayesian Reciprocator uses a probabilistic and generative approach to infer the latent preferences, beliefs, and strategies of others through interaction and observation. We evaluate the Bayesian Reciprocator using a generator over games where every interaction is unique, as well as in classic environments such as the iterated prisoner’s dilemma. The Bayesian Reciprocator enables the emergence of both direct-reciprocity when games are repeated and indirect-reciprocity when interactions are one-shot but observable to others. In an evolutionary competition, the Bayesian Reciprocator outcompetes existing automata strategies and sustains cooperation across a larger range of environments and noise settings than prior approaches. This work quantifies the advantage of a theory of mind for cooperation in an evolutionary game theoretic framework and suggests avenues for building artificially intelligent agents with more human-like learning mechanisms that can cooperate across many environments.

## Significance

Theory of mind is the ability to understand other people’s behavior in terms of mental states such as desires and beliefs. Many have hypothesized that theory of mind is important for explaining the distinct scale, scope, and sophistication of human cooperation. However, there is still an open question of how theory of mind actually leads to enhanced cooperation. Here, we develop a computational model of theory of mind and use it to develop an agent that conditionally cooperates with agents it infers are like itself. In evolutionary game theoretic simulations, our agent leads to the emergence of cooperation in a wider range of games and outcompetes less sophisticated agents that lack a theory of mind.

Explaining the evolution of cooperation—where self-interested individuals pay costs to create collective benefits—has been a central focus of research across the natural and social sciences for decades (1–6). A key conclusion that has emerged from this work is the centrality of reciprocity in human cooperation: evolutionary game theoretic models demonstrate that direct reciprocity (I’ll help you if you help me) is possible when interactions between individuals are repeated (1, 2, 7–10) and indirect reciprocity (help those who help others) is possible when one-shot interactions are witnessed by observers and individuals keep track of reputations (11–17). Surprisingly simple automata models of reciprocal interactions in evolutionary games such as an iterated prisoner’s dilemma (IPD) or donation games have provided elegant accounts of how conditional cooperation can arise between unrelated individuals and revealed fundamental insights into the behavioral mechanisms necessary to sustain it. For example, in repeated games, strategies such as tit-for-tat (TFT) and win-stay-lose-shift (WSLS) (8) begin by acting cooperatively but retaliate when defected upon in order to punish and discourage cheaters who would exploit the altruism of other agents. However, the simplicity of both the environments and models imposes stark limits on the generality of these accounts, especially if taken as accounts of human cooperation.

First, human interactions are almost infinitely varied and not restricted to a single game with a fixed number of players and decisions (2 players, 2 actions in PD), while most automata are defined only for a single kind of specific (if stylized, generic) game such as IPD. For automata, even small variations in the environment, such as the degree of noise (7, 9, 15), variable payoffs (18–21), whether actions are made simultaneously or sequentially (22–25), the number of actions available (26, 27) or whether players can observe the actions of others (13, 14), all require different strategies. Yet even when the same two people repeatedly interact in the same context, no two interactions have exactly the same payoff structure; more broadly, we engage in all manner of different interactions across which the number of participants, the options available to each participant, and the resulting payoffs differ markedly (and often unpredictably). Because of this variation, it is implausible (and impractical) to imagine that human beings have learned or evolved a new strategy for every possible game they might encounter. Rather than a specific strategy for each specific game, human cognition supports general cooperative strategies that can be applied anywhere (28–30).

Second, in contrast to standard automata that operate only at the behavioral level, people make predictions about the cooperative potential of partners based on inferences over the latent (unobservable) intentions, motives, and traits that underlie those agents’ behavior (31, 32); taking into account the possibility that observed actions may only noisily reflect agents’ intentions or traits allows us to robustly handle the uncertainty inherent in complex social interactions in a complex dynamic world (33). People learn about cooperative partners and their motives by integrating across long histories of interactions into mental models of others, both their own experiences and by observing third parties, not just their most recent behavior as standard automata do. In humans, the capacity to make inferences about these latent intentions and traits from sparse and noisy observations of behavior is a crucial part of our “theory of mind” (34, 35). Theory of mind is thought to be present in young children, and in some more limited form even in preverbal infants, playing an important role in how we develop a sense of prosocial norms and moral judgment (36–39).

This paper introduces the Bayesian Reciprocator, an approach to modeling the evolution of human cooperation that highlights the value of rational theory of mind inferences in supporting agents’ robust cooperation across a large class of environments and settings. Our approach brings together key ideas for modeling humans that have been highly influential in cognitive science, economics, and computer science. First, subjective utility functions that express general preferences (as opposed to game-specific behavioral rules) enable generalizable decision making that is sensitive to the payoffs and structure of new games (40). Second, the potential to value the payoffs received by other cooperative players in one’s own utility function produces generalizable cooperative and altruistic behavior (41–43). Third, dynamically adjusting how others are valued proportional to one’s belief that they are cooperating in the same way, realizes a powerful form of reciprocity based on shared values (44–46). Fourth, Bayesian inference over a generative model of the latent decision-making and learning processes of others, Bayesian theory of mind, enables players to rapidly and robustly infer the utility functions of others under uncertainty and noise, thereby identifying the cooperators that they, in turn, should cooperate with (47–51). In short, the Bayesian Reciprocator conditionally cooperates with a kind of virtue ethics; the reputation (and worthiness as a cooperative partner) of other players is determined by their latent utility function, which is revealed through behavior (52, 53). Finally, the Bayesian Reciprocator unifies many key features that have been shown to be important for cooperation: reciprocity, reputation, relationships, robustness under noise, forgiving of errors, and is grounded in the computations of the earliest emerging and most distinctly human cognitive operations: utility-based decision making, probabilistic inference, and theory of mind.

**Fig. 1.**
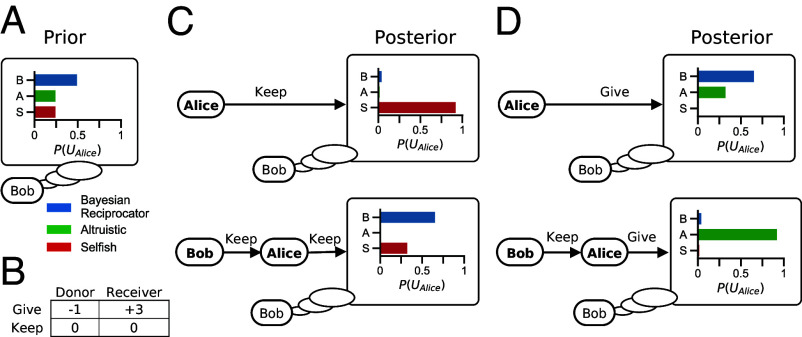
Belief dynamics with a Bayesian theory of mind. (*A*) Beliefs over other player’s utility functions are represented as probability distributions. Shown is Bob’s prior over Alice’s utility function ($U_{\mathrm{Alice}})$. (*B*) Payoffs for the Give-Keep game. The donor decides whether to pay 1 and give 3 to a receiver (“give”) or do nothing (“keep”). (*C* and *D*) Bob’s posterior over *U*_*Alice*_ resulting from inferences from Alice’s behavior after a short interaction. The same action by Alice can lead to different beliefs depending on the prior interaction. If Alice chooses keep Bob infers she is likely Selfish (*C*, *Top*), but if Alice chooses keep after Bob also played keep, she is inferred to be either Bayesian Reciprocator or Selfish as her action can be interpreted as reciprocation (*C*, *Bottom*). If Alice chooses give (*D*, *Top*), Bob’s beliefs update to put higher weight on her either being Bayesian Reciprocator or Altruistic and lower probability of her being Selfish, but if Alice chooses give after Bob played keep she is inferred to be Altruistic (*D*, *Bottom*).

**Fig. 2.**
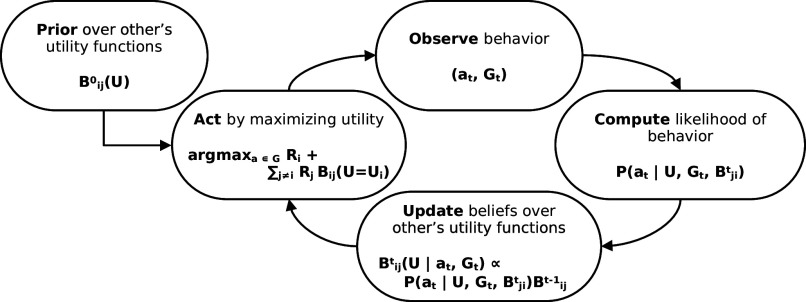
The action, observation, learning loop for the Bayesian Reciprocator. Start with prior beliefs over the utility functions of the other players. Act according to those beliefs and observe the actions of others. Calculate the likelihood of that behavior given the different models in the prior and use them to update the posterior. Finally, act in the next game, and the learning loop repeats.

**Fig. 3.**
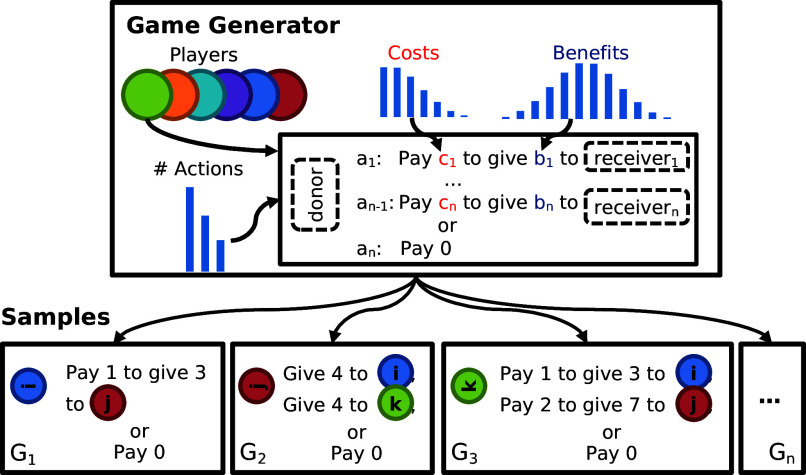
The Game Generator. Instead of a single game repeated many times, the Game Generator creates an infinitude of social choices where the number of actions available to the decision making player, the costs, and benefits to each affected player are sampled probabilistically. The Game Generator is parameterized by a set of players (colored circles), distributions over the number of costs, benefits, and actions composed in a template. Each sample $\left(G_{1},\ldots ,G_{n}\right)$ from the Game Generator is a unique social decision making problem. The rotated player on the left (e.g., player i, in *G*_1_) is the decision maker, and they are given a set of options that depend on the sampled values of *b* and *c*. Each sampled decision includes the action “Pay 0” where no costs or benefits are distributed to any player.

**Fig. 4.**
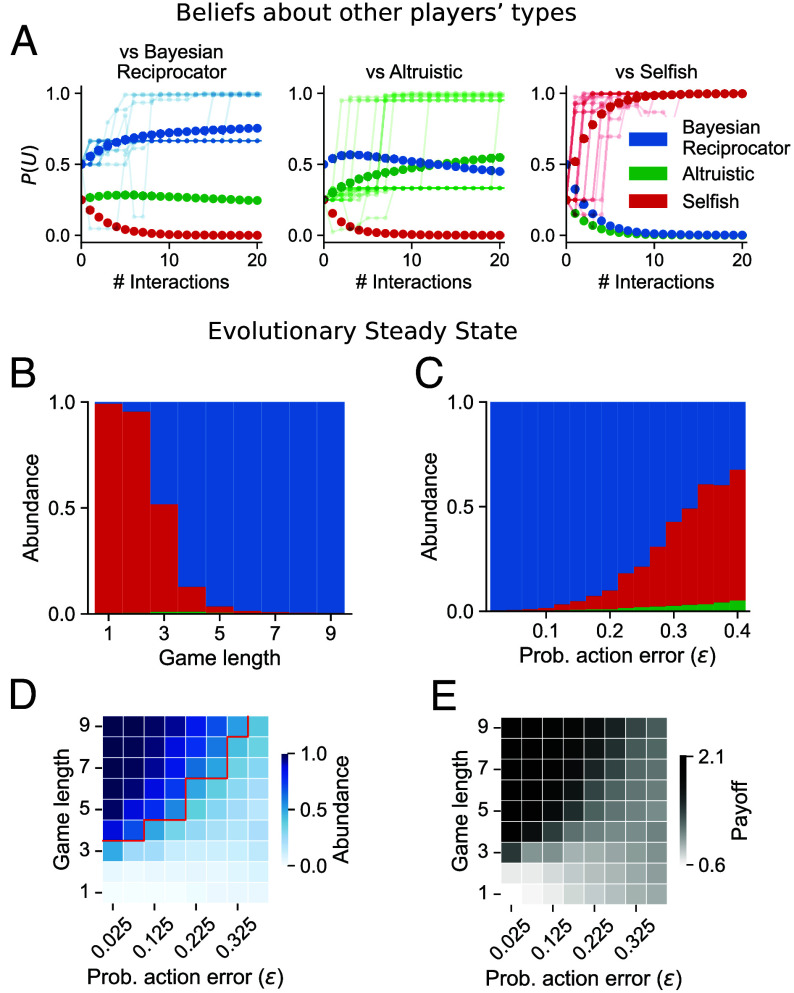
The Bayesian Reciprocator learns about others from sparse data during interaction, leading to robust cooperation in the Game Generator. (*A*) Intragenerational belief updating. In each case, a probe Bayesian Reciprocator was matched with another Bayesian Reciprocator (*Left*), an Altruistic player (*Center*), or a Selfish player (*Right*), and beliefs were monitored for 20 interactions. After each interaction, the beliefs of the probe player were measured. Dark solid points show an average of over 1,000 trials. Faint traces are single belief trajectories. In all cases, beliefs move from the prior (0 pairwise interactions) toward correct beliefs but at different rates. Bayesian Reciprocator quickly learns when paired with a Selfish player but has a harder time distinguishing Bayesian Reciprocator from a Altruistic player. (*B*–*E*) Intergeneration evolutionary steady state of the population in the repeated Game Generator under the Moran process while varying (*B*) the length of the repeated game in each generation or (*C*) the probability of an error during action selection. The size of each bar color is the steady state proportion of the population with the player type of that color. (*D*) The abundance of Bayesian Reciprocator in the steady state population for different amounts of repetition and action error. Above the red line, the Bayesian Reciprocator proportion is greater than 0.5 of the steady-state population. (*E*) Population payoffs for different error rates and game lengths. Higher abundance of the Bayesian Reciprocator yields higher payoffs due to cooperation.

**Fig. 5.**
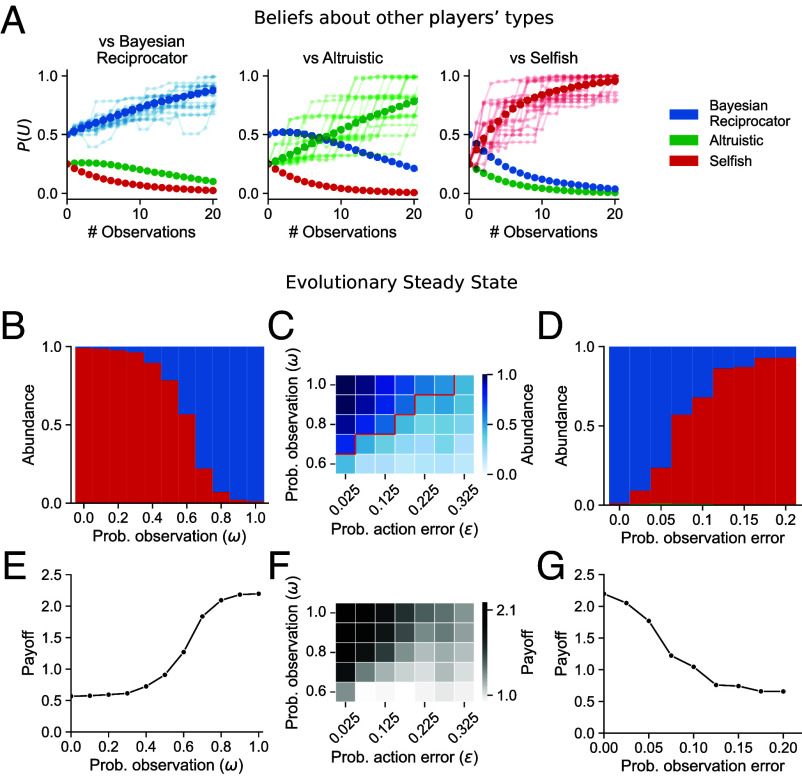
The Bayesian Reciprocator learns from observation and robustly cooperates even when players only interact once. (*A*) Intragenerational belief updating in a population of 10 players where all players observe each other’s actions. Beliefs are shown after each observation for each player type: Bayesian Reciprocator (*Left*), Altruistic (*Center*), or Selfish (*Right*). Dark solid points show an average of over 1,000 trials. Faint traces are single belief trajectories. (*B*) Intergeneration evolutionary steady state (Moran process) of the population in the one-shot Game Generator while varying the probability (*ω*) that each action is observed by all players or only those involved in the interaction (1−ω). Players only interact with each other once. The size of each colored bar is the proportion of the population with the player of that color. (*C*) The Bayesian Reciprocator is robust to action errors in this one-shot setting, but higher error rates require a greater fraction of interactions observable by all. (*D*) In contrast, the Bayesian Reciprocator is more sensitive to perception errors. (*E*–*G*) Population payoffs for the scenarios (*B*–*D*) show that a higher abundance of the Bayesian Reciprocator leads to higher levels of cooperation.

**Fig. 6.**
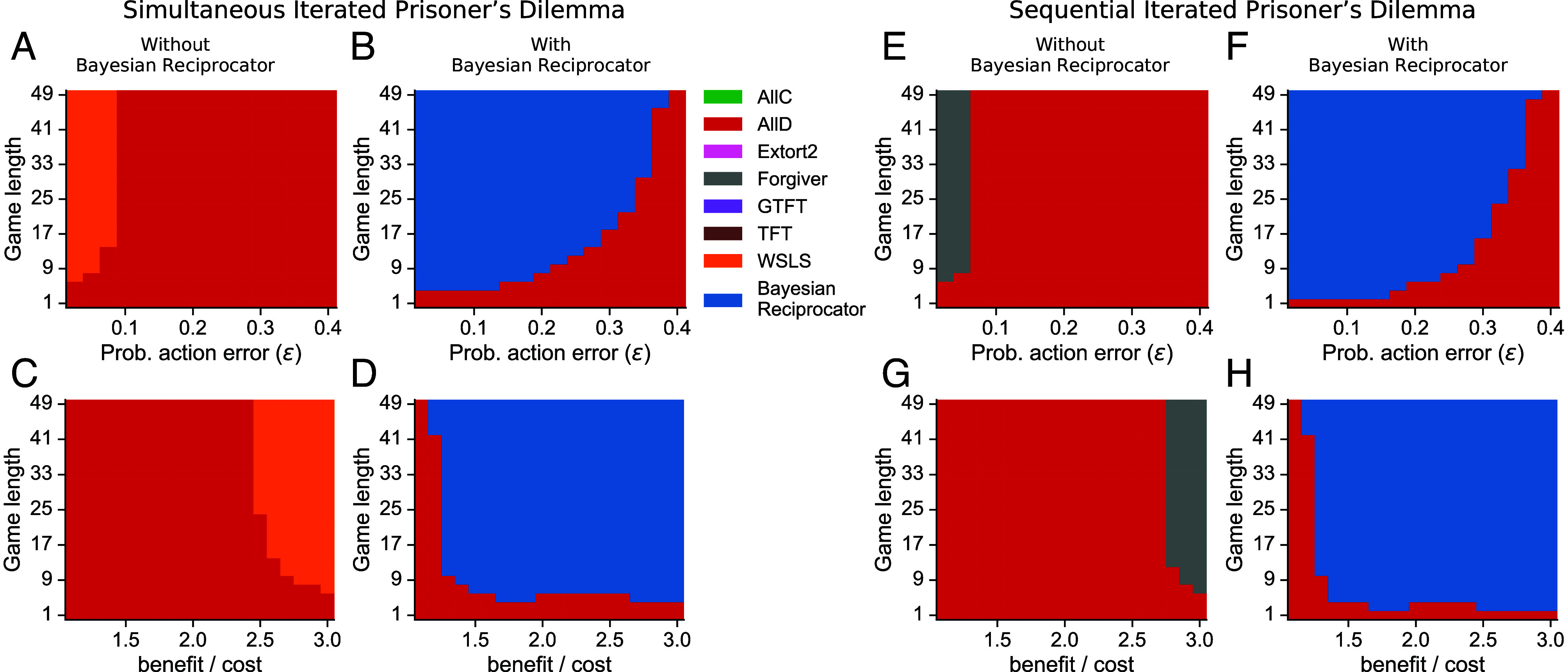
The Bayesian Reciprocator outcompetes leading automata strategies and expands the range of environments where cooperation is possible in simultaneous (*A*–*D*) and sequential (*E*–*H*) IPD. We analyzed the most prevalent strategy with and without the Bayesian Reciprocator while varying two pairs of parameters: the probability of action error and game length (*A*, *B*, *E*, and *F*) and the benefit/cost ratio and game length (*C*, *D*, *G*, and *H*). Without the Bayesian Reciprocator present, WSLS (yellow; *A* and *C*) and Forgiver (gray; *E* and *G*) are the most prevalent strategies in the simultaneous and sequential IPD, respectively. However, AllD (red) was most prevalent for all but the lowest error rates and highest benefit/cost ratios. When the Bayesian Reciprocator was added to the evolutionary simulation, it outcompetes the automata strategies for most parameter pairs (blue) in both the simultaneous (*B* and *D*) and sequential (*F* and *H*) versions of the IPD. Furthermore, the Bayesian Reciprocator enables cooperation for much higher error rates and lower benefit/cost ratios than the automata strategies alone.

We first describe the Bayesian Reciprocator and describe its learning and decision-making dynamics (Figs. 1 and 2). Next, we develop a setting for studying the evolution of cooperation, the Game Generator, where every interaction between players is unique and varies in terms of the number of players, the number of actions, and the payoffs (Fig. 3). Using evolutionary simulations, we show that the Bayesian Reciprocator achieves cooperative equilibria in the Game Generator through both direct and indirect reciprocity (Figs. 4 and 5). Finally, we show that the Bayesian Reciprocator outcompetes common automata strategies in the IPD and expands the scope of cooperation in that game (Fig. 6).

## The Bayesian Reciprocator

A game *G* is a set of actions *a* that specify positive and negative payoffs R(a) for each player. Player *i* from a population *N* players has payoff $R_{i}\left(a\right)$ and a private subjective utility function $U_{i}\left(a\right)$. Players with a utility function (as opposed to automata strategies) select the action ($a^{*}$) with the highest (expected) utility with ties broken randomly.

[1] $$\begin{array}{c} a_{i}^{*}=\underset{a\in G}{\arg\;\max}U_{i}(a). \end{array}$$

All players experience action errors where a nonintended randomly chosen action is taken instead of the player’s intended action with probability *ϵ*. When these errors happen, only the implemented action is observed; the intention is not.

Players track their beliefs about the utility functions used by others as $B_{\mathrm{ij}}\left(U\right)$, which represents the degree of belief ([0,1]) player *i* has that player *j* is using utility function *U*. The Bayesian Reciprocator is defined by the utility function (action dependence dropped for clarity):

[2] $$\begin{array}{c} U_{i}=R_{i}+\sum_{j\neq i}R_{j}*B_{\mathrm{ij}}\left(U=U_{i}\right). \end{array}$$

That is, the Bayesian Reciprocator subjectively values their own payoff (*R*_*i*_) plus the payoffs received by other players, ($R_{j\neq i}$) proportional to their own belief that each other player *j* has the same utility function $B_{\mathrm{ij}}\left(U=U_{i}\right)$. When $B_{\mathrm{ij}}\left(U=U_{i}\right)$ approaches one, the Bayesian Reciprocator follows the maxim of treating others as it would treat itself (54), when $B_{\mathrm{ij}}\left(U=U_{i}\right)$ approaches zero, the Bayesian Reciprocator has no regard for the payoff of the player and acts only toward its direct interests. Thus, the Bayesian Reciprocator is a parochial cooperator. It narrowly values the welfare of only those it recognizes as the same type (55).

In this work, we study abstract resource distribution and trade-off games. However, realistic decisions and, thus, realistic utility functions will inevitably involve choosing between diverse goods and currencies. For instance, players may have arbitrary preferences over foods, humor, activities, work, etc. While we will not delve into this additional complexity formally, our intention is that the comparison of utility functions, $B_{\mathrm{ij}}\left(U=U_{i}\right)$, include only the part of the utility function that concerns the valuation of others’ welfare. It is interesting to consider that including personal preferences as a condition for cooperation may lead to moralization, in/out-group effects, or polarization.

We will primarily analyze the evolution of the Bayesian Reciprocator in the presence of two other utility functions: a Selfish player that values only its own payoff ($U_{i}=R_{i}$) and an unconditionally Altruistic player that values its own payoff equally with all other players ($U_{i}=R_{i}+\sum_{j\neq i}R_{j}$). These utility functions generalize the unconditional automata Always Defect (AllD) and Always Cooperate (AllC). The utility functions of all players are always private and thus not observable by any other player. Thus the Bayesian Reciprocator leverages theory of mind, the ability to infer the latent causal forces that drive action (in this case, their utility functions) from their behavior.

To carry out these inferences, we draw the idea that Theory of Mind, can be modeled as Bayesian inference over a generative model of another agent. Bayesian theory of mind (BToM) has shown success at modeling empirical human judgments of mental states across a wide variety of contexts: attributing beliefs and desires to a single agent making decisions under uncertainty (56), judging whether an individual is helping (or hindering) another (48), whether a group is working together or competing with each other (49–51), and how to coordinate with communication (57). It has also been influential in developing agents that understand the actions of other agents and people (58–60).

Applying BToM to a two-player setting without loss of generality, when player *i* observes *j*’s action $a_{j}^{t}$ in $G^{t}$ at time *t*, then *i* updates its previous beliefs $B_{\mathrm{ij}}^{t-1}$ to $B_{\mathrm{ij}}^{t}$ following Bayes rule:

[3] $$\begin{array}{c} B_{\mathrm{ij}}^{t}\left(U|a_{j}^{t},G^{t}\right)\propto P\left(a_{j}^{t}|U_{j},G^{t},B_{\mathrm{ji}}^{t}\right)B_{\mathrm{ij}}^{t-1}\left(U|a_{j}^{t-1},G^{t-1}\right), \end{array}$$

the likelihood, $P(a_{j}^{t}|U_{j},G^{t},B_{\mathrm{ji}}^{t})$ is the probability of the *j*’s action $a_{j}^{t}$ at time *t* in game $G^{t}$ assuming they have the utility function *U*_*j*_ and beliefs about *i*’s utility function $B_{\mathrm{ji}}^{t}$. This probability is equal 1−ϵ for the action chosen by Eq. **1** and equal to ϵ/|G| (where |G| is the number of actions) for each other action in the game to account for action error.

The iterative update of $B^{t}$ based on $B^{t-1}$ terminates at *t* = 0 which is the prior, $B_{\mathrm{ij}}^{0}\left(U\right)$. The utility functions and player types in $B_{\mathrm{ij}}^{0}\left(U\right)$ with nonzero probability determine the support for inference. To encourage prosocial initial behavior in the Bayesian Reciprocator, the prior on others sharing the same type is $B_{\mathrm{ij}}^{0}\left(U_{j}=U_{i}\right)=0.5$, and the remaining probability is distributed equally over the other utility functions and player types included in the evolutionary tournament (Fig. 1*A*). Varying the prior weight placed on $B_{\mathrm{ij}}^{0}\left(U_{j}=U_{i}\right)$ shows that our results are robust to the specific value chosen (*SI Appendix*, Fig. S1). Our inferential framework could be extended to include learning this prior, but we leave this extension for future work (61).

However, computing the likelihood in Eq. **3** requires *i* to know the beliefs that *j* has about *i*, $B_{\mathrm{ji}}^{t}$. These beliefs are also private and must be inferred. Prior work on BToM analyzed a setting where an external third-party observer is watching the behavior of one or more agents and does not consider the challenge of multiple agents, each with a BToM interacting with and learning about each other. A recursive formulation of theory of mind is needed as the target of inference is making its own inferences. In children, the capacity for these higher-level inferences about what others think emerges early and has been shown to influence social behavior and moral judgment (62, 63).

Most importantly for cooperation, without a recursive theory of mind, players will not be able to distinguish a justified withholding of cooperation (e.g., a reciprocal response to defection) from unjustified selfish withholding of cooperation. The difference between these two actions depends on an inference about the actor’s beliefs: was the actor a Bayesian Reciprocator but with the belief that the recipient is not, was the actor simply a Selfish player who always acts selfishly, or an Altruistic player who tried to cooperate but withheld cooperation due to an action error? Each of these hypotheses could explain the ambiguous action to a variable degree and must be quantified correctly.

One approach to a recursive theory of mind is to have each player model each other’s beliefs in an (in)finite regress: each player must track “what Alice knows, Bob knows Alice knows...” and so on (64). In practice, this kind of regress is approximated with a finite number (K) of nested models that ground out in nonlearning (K = 0) model (65–67). However, as approximate models, they lead to unstable and divergent beliefs and require great computational cost (68). Even when possible, the number of models (and hence belief updates) required grows exponentially. Alice would need to model Bob, modeling Carl, modeling Alice, and so on.

Instead, we develop a method for efficiently tracking recursive beliefs when players believe they have common knowledge that they observed the same interaction as other players (69–71). The basic intuition is that instead of tracking a hierarchy of beliefs, the Bayesian Reciprocator tracks each subset of common knowledge (72, 73). Under this formulation, Alice’s model of Bob’s beliefs is identical to the beliefs an external player in a “view from nowhere” would form had they observed what was observed by both Alice and Bob (74). Formally, the likelihood of *j*’s action as perceived by player *i* in Eq. **3** can be written as $P\left(a_{j}^{t}|U_{j},G^{t},B_{\mathrm{ji}}^{t}\right)=P\left(a^{t}|U_{j},G^{t},B_{j\cap i}^{t}\right)$, where $B_{j\cap i}^{t}$ denotes the belief state conditioned on the common observations of players *i* and *j* up to time *t*.

Because j∩i=i∩j, players do not need to represent a growing hierarchy of recursively nested models. This approach to belief representation greatly simplifies reasoning about social knowledge when a large group of players observe new information. Instead of updating what each player believes individually (and what they believe others believe and so on), this representation allows players to directly update the beliefs of each group of players all at once. See *SI Appendix*, *Supplementary Methods* for algorithmic details on the implementation of this belief update.

Fig. 1 shows an example of how two Bayesian Reciprocator update their beliefs in a simple two-action game where players sequentially choose whether or not (give or keep) to pay a small cost to provide a larger gain to another player over two-time steps. Players combine their prior beliefs (Fig. 1*A*) with the information a new observation provides about the utility function that generated that behavior (Fig. 1 *C* and *D*). Simple reciprocal behavior emerges from the utility function of the Bayesian Reciprocator (Eq. **2**) and the learning dynamics of Bayesian inference (Eq. **3**). If Alice does not cooperate, Bob’s belief about her utility function shifts to highly weight the Selfish type, which leads to Bob withholding cooperation. Finally, Fig. 2 shows how these inferences are combined into a learning loop that continues for the duration of a repeated game.

## The Game Generator

To test the generality of the Bayesian Reciprocator across many game types, we developed a game theoretic setting called the Game Generator. The Game Generator is a probabilistic generative process that uses a general resource allocation template to create an infinite number of distinct cooperation challenges. The template is shown in Fig. 3. In each sample, a donor is selected who can then opt to transfer resources to one or more receivers. These transfers may be costly or costless. Many familiar games, such as the prisoner’s dilemma, altruistic giving games where players can give up some of their own welfare to help another person, allocation games where players can show favoritism in choosing who should receive an indivisible resource, and even moral dilemmas where players bear no personal costs themselves but decide outcomes for groups of others are unified under this sampling process.

The Game Generator can create repeated games by having the same pair of players play a number of samples together (game length). Other parameters such as the average costs (*C*), benefits (*B*), number of actions per sample, probability of action error (*ϵ*), action observability (*ω*), and observation error are all controllable knobs. No two interactions sampled from the Game Generator are ever exactly alike. Actions are randomly ordered and lack semantic labels, so all decisions and inferences must be made in terms of the sampled payoffs (costs and benefits). Due to variation in both the payoffs and the number of actions of each sampled game, traditional automata-based strategies cannot be directly applied to the interactions sampled from the Game Generator. See Fig. 3 and *SI Appendix* for the details of the generative process and some example generated games.

## Results

### Direct Reciprocity in the Game Generator.

We first study the evolution of cooperation through direct reciprocity in the Game Generator environment with the Bayesian Reciprocator, Selfish, and Altruistic players. To study direct reciprocity, we used the Game Generator to generate varied repeated interactions between players where interaction between players are private (*ω* = 0), i.e., only observable to the actor and the player(s) that could have received a resource.

To better understand the behavior of the Bayesian Reciprocator in the context of multiple repetitions, we first analyzed the dynamics of belief during a repeated interaction. Fig. 4*A* shows the average beliefs formed by a Bayesian Reciprocator after 20 repeated interactions with either another Bayesian Reciprocator, an Altruistic player, and a Selfish player. Over the course of the repeated interactions, the Bayesian Reciprocator’s beliefs update to correctly distinguish between other Bayesian Reciprocator, Altruistic, and Selfish players. Importantly, the Bayesian Reciprocator identifies Selfish players rapidly (often after just a few interactions), which is necessary for conditional cooperation. The Bayesian Reciprocator learns to distinguish Altruistic players from the Bayesian Reciprocator more slowly as both are initially cooperative. These belief updates happen within a generation.

Next, we looked at evolutionary success across generations by characterizing the steady state distribution of players under the Moran process (75, 76). Under the Moran process, a mutant player type can invade if it is neutral or even at a disadvantage (77). These invasions act as stepping stones between player types and can cause cycles in the population composition (78, 79). Thus, we show the relative abundances of the different strategies at steady state rather than only presenting the most prevalent player type. Experiments were carried out in a population of 10 players, with a small probability of action errors (*ϵ* = 0.025), and parameters *B* = 5, *C* = 1. See *SI Appendix* for details of the steady state distribution calculations.

Fig. 4*B* shows the steady state distribution as a function of the game length, the number of samples from Game Generator played by each pair of players. When the game length in each generation is short (<3), the Selfish player outcompetes the Bayesian Reciprocator and the Altruistic player. As the probability of repetition increases (≥3), the Bayesian Reciprocator becomes the most prevalent strategy in the population. We next analyzed the evolutionary steady state while varying the probability of action error with a game length of nine rounds. Fig. 4*C* shows that the Bayesian Reciprocator is robust to noise and outcompetes the Selfish player when the error rate is <0.3.

The higher the probability of an action error, the longer the game length required for the Bayesian Reciprocator to enable cooperation (Fig. 4*D*). Higher error rates are challenging because they slow down learning, and thus, longer game lengths are needed to identify the types of others. Fig. 4*E* shows that the Bayesian Reciprocator is actually finding a cooperative equilibrium that improves the joint payoffs of the population compared to the Selfish player. In the parameter regions where the Bayesian Reciprocator is the most prevalent strategy in equilibrium, the average population payoffs are also high. Together, these results show that the Bayesian Reciprocator forms directly reciprocal relationships leading to the evolution of cooperation in noisy and variable environments.

Finally, we also show that the Bayesian Reciprocator also outperforms players with more sophisticated utility functions in the repeated Game Generator. In addition to the Selfish and Altruistic players, the model also outcompetes an inequality averse player that tries to keep its cumulative payoffs balanced with its partners (80). See *SI Appendix*, Fig. S2 for results and implementation details.

### Indirect Reciprocity in the Game Generator.

Having established the evolution of cooperation under conditions favoring direct reciprocity, we next studied the evolution of cooperation in the Game Generator but where players were never matched with the same player more than once (game length = 1), making it impossible for a player to form a directly reciprocal relationship with another. Instead, we varied the probability of observation (*ω* ≥ 0), which allows players to observe the behavior of others even when they are not involved in the decision themselves. This setting allows us to study the evolution of cooperation through indirect reciprocity. The Bayesian Reciprocator requires no modification to its structure or parameters for this setting. Mathematically, inference from one’s own interactions or from observing the interactions of others is just conditioning on a different source of data.

As before, we first study the intragenerational learning dynamics in a population of 10 players with four Bayesian Reciprocator three Selfish players and three Altruistic players. Players interact with each other no more than once, but all interactions are observable by all other players (*ω* = 1). Fig. 5*A* shows that Bayesian Reciprocator rapidly learns the true type of each player from sparse observations. In all cases, beliefs move from the initial prior (0 observations) toward the correct belief. When comparing the dynamics of learning here to learning from repeated interactions (Fig. 4*A*), learning from observation enables the Bayesian Reciprocator to more rapidly distinguish the Bayesian Reciprocator and Altruistic players since Altruistic players will be observed unconditionally cooperating with known Selfish players.

We next studied the evolution of cooperation through indirect reciprocity in the Game Generator environment with Bayesian Reciprocator, Selfish, and Altruistic players. All experiments were carried out with the same parameters as before, but we set game length to 1 and varied observability (*ω*). As expected, when the probability of observation was low, the Selfish player was the most prevalent strategy as there is no consequence for uncooperative behavior, and players cannot reliably learn the types of others. As the probability of observation grows, the Bayesian Reciprocator becomes the most prevalent player in the population (Fig. 5*B*). This transition from Selfish to Bayesian Reciprocator was accompanied by a jump in the population payoffs, showing that the Bayesian Reciprocator enables the evolution of cooperation through indirect reciprocity.

Next, we assessed the robustness of indirect reciprocity via Bayesian Reciprocator under action and observation errors. Unlike action errors, where a more cooperative choice might be stochastically replaced with a less cooperative choice (accident), observation errors are challenging because players will occasionally be exposed to different data and form divergent beliefs even though each player will believe they have seen the same data as others (15, 81). Fig. 5*C* shows the model is highly robust to action errors. While higher action error rates require a great percentage of observations to be observable to all, cooperation can still be sustained with an error rate of over 0.20. For observation errors, Fig. 5*D* shows that indirect reciprocity driven by the Bayesian Reciprocator is robust to small amounts of observation errors (for *ω* = 1). When the perception error rate reaches 0.075 and above, the Selfish player outcompetes all others at steady-state. Fig. 5 *F* and *G* show that as long as the Bayesian Reciprocator is the most prevalent player at steady state in both error models, the cooperation rate (as measured by total payoff) stays high.

Finally, we show that without modification, the model can integrate repeated interactions and observability, allowing for a mixture of direct and indirect reciprocity. This is a more realistic setting for human cooperation where both of these forces are often present simultaneously. *SI Appendix*, Fig. S3*A* shows the abundance of the Bayesian Reciprocator in the steady state while varying both the probability of observation and the game length. Empirically, we find a roughly linear relationship between game length and observability in the Game Generator environment, suggesting that both direct and indirect reciprocity can mutually support each other (*SI Appendix*, Fig. S3*B*). However, they may do so in ways that are independent.

### IPD.

While the Bayesian Reciprocator enables the emergence of robust direct and indirect reciprocity in the Game Generator, in that setting, we could not directly compare agents against classic automata strategies. The classical automata require two action games where the cooperative and noncooperative actions are labeled (unlike in the dynamic Game Generator environment). Therefore, we investigated the performance of the Bayesian Reciprocator in the sequential and simultaneous IPD where there are already well-established successful strategies. Specifically, we compared Bayesian Reciprocator to AllD, AllC, TFT (82), generous TFT (GTFT) (7), WSLS (8), Forgiver (24), and against the more recently developed extortion strategies (83, 84) (see *SI Appendix* for details on these automata).

In the simultaneous variant of the IPD, both players choose whether to cooperate or defect, and only after both players have chosen are the actions observed and payoffs received. In the sequential variant of the IPD, players choose sequentially, and actions are observed as soon as they are made. We choose to study both the sequential and simultaneous versions of the IPD because a different set of automata succeed in each variant. In the simultaneous IPD, WSLS is most prevalent, but in the sequential IPD, Forgiver is most prevalent (24).

We ask whether the Bayesian Reciprocator can allow cooperation to evolve in parameter regions where AllD outcompetes the cooperative automata. To do so, we first search through parameter space for cooperative equilibria when the Bayesian Reciprocator is not included in the simulation. Then, we do the same search but with the Bayesian Reciprocator. We first varied execution errors along with game length in the simultaneous (Fig. 6 *A* and *B*) and sequential (Fig. 6 *E* and *F*) IPD. When the error rate is high or the game length is short, AllD is most prevalent (shown in red) whether or not the Bayesian Reciprocator was included. When both the error rate is low and the game length is long, both the automata strategies alone (WSLS in simultaneous IPD; yellow, Forgiver in sequential IPD; gray) and together with the Bayesian Reciprocator yield cooperative equilibria. But for higher error rates or shorter game lengths, the presence of the Bayesian Reciprocator is required for a cooperative equilibrium to emerge (blue).

We find similar results when varying the benefit/cost ratio in the simultaneous (Fig. 6 *C* and *D*) and sequential (Fig. 6 *G* and *H*) IPD. For the lowest benefit/cost ratios and shortest game lengths, AllD is the most prevalent strategy (red). For high benefit/cost ratios, both the automata strategies alone (WSLS in simultaneous IPD; yellow, Forgiver in sequential IPD; gray) and with the Bayesian Reciprocator yield cooperative equilibria. However, for an intermediate benefit/cost ratio closer to one, the presence of the Bayesian Reciprocator is required for a cooperative equilibrium to emerge (blue). For nearly all parameter pairs tested, when the Bayesian Reciprocator is included with the automata strategies, the most prevalent strategy at steady state is the Bayesian Reciprocator (Fig. 6*B*, *D*, *F*, and *H*).

**Fig. 7.**
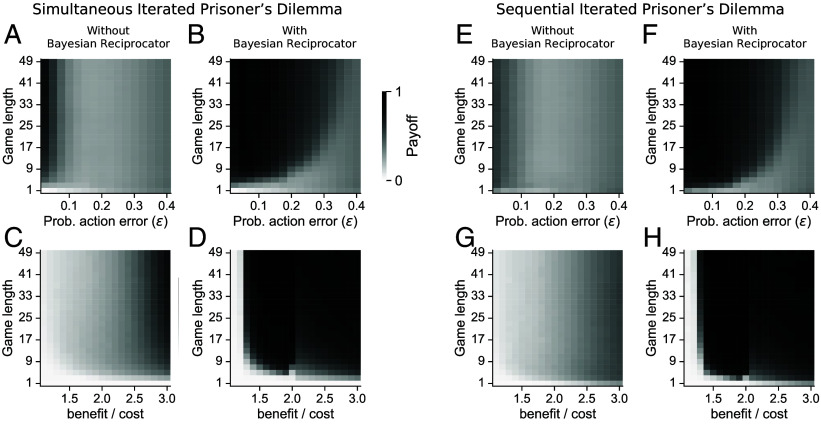
The Bayesian Reciprocator widens the range of parameters that enable cooperation in the simultaneous (*A*–*D*) and sequential (*E*–*H*) IPD. The population of players is the same as Fig. 6. Heatmaps show the average population payoff at steady state (normalized between 0 and 1). Without the Bayesian Reciprocator, cooperation (darker gray regions that correspond to higher population payoffs) is limited to long game lengths, low probabilities of action errors, and high benefit/cost ratios (*A*, *C*, *E*, and *G*). With the Bayesian Reciprocator, cooperation emerges across a wider range of environmental parameters and the cooperation that does occur is closer to the maximum, i.e., the gray regions are darker (*B*, *D*, *F*, and *H*).

In each case, for parameter regions where the Bayesian Reciprocator is the most prevalent player, the average population payoff (i.e., cooperation rates) is higher (Fig. 7). *SI Appendix*, Fig. S4 shows the relative abundance of each player type at steady state. Although some automata are present in small amounts, the Bayesian Reciprocator accounts for the majority of the time spent in a cooperative state. Finally, these same results hold when we allow for all deterministic memory-1 strategies to compete against the Bayesian Reciprocator (24). The Bayesian Reciprocator leads to more cooperative behavior and thus higher populations payoffs for a wider range of parameter settings (*SI Appendix*, Fig. S5) and at steady-state is the most prevalent strategy when the population is cooperating (*SI Appendix*, Fig. S6).

Taken together, these results show that in the IPD, the Bayesian Reciprocator expands the range of cooperative equilibria compared to the leading automata strategies. This was seen in both low benefit/cost and high error rate environments, which might be particularly important for cooperation to get off the ground. Finally, while different cooperative automata were most prevalent at steady state in the simultaneous and sequential IPD, the exact same parameterization of the Bayesian Reciprocator was consistently the most prevalent strategy across both variants of the IPD. Unlike the existing strategies that have been hand-engineered for cooperation in the IPD over many decades, the Bayesian Reciprocator is a general cooperator that excels even in the special case of the IPD in addition to the more general Game Generator.

## Discussion

We introduced the Bayesian Reciprocator, a model for the evolution of cooperation based on insights from artificial intelligence and the computational study of human social cognition. The Bayesian Reciprocator recursively values the rewards of others proportional to its belief that others are cooperating in the same way. These beliefs are updated through interaction with and observation of others by a recursive and Bayesian model of theory of mind. We demonstrated the value of this approach in an environment that is much richer than games typically studied, the Game Generator, where every decision is a sample from a generative model so that players never make the same decision twice, and decisions and judgments must be made in terms of their outcomes and alternatives. Using evolutionary simulations, we show that the Bayesian Reciprocator enables the evolution of cooperation in the Game Generator both through direct reciprocity when interactions are repeated but private and through indirect reciprocity when interactions are one-shot but publicly observable. Finally, the Bayesian Reciprocator outperformed existing automata strategies and expanded the range of cooperative equilibria when applied in the context of the IPD game.

Together, these results show the power of a cognitively sophisticated strategy in general—and theory of mind in particular—for enabling robust cooperation. Deeper still, these studies may explain how the benefits of cooperation may have driven the evolution and emergence of theory of mind. Theory of mind explains how and why general and robust cooperation can evolve—but also, cooperation might explain how and why theory of mind evolved and became so important in human cognition. In the spirit of the “cognitive niche” and “cultural niche” accounts of human evolutionary success (28, 85), social reasoning abilities such as theory of mind may coevolve with other aspects of distinctively human sociality: capacities for general and flexible cooperation, learning socially from others, and cumulative culture (86). Our simulations quantify how sophisticated social reasoning of this type delivers cooperative benefits above and beyond what can be achieved by less cognitively flexible agents—and thus can outcompete simpler strategies evolutionarily.

The Bayesian Reciprocator has a number of properties desirable for conditional cooperation that emerge from the recursively dependent utility function and the ability for players to infer the latent utility functions of others from their actions. First, the model operates with a sophisticated and realistic reputation system: cooperators punish (by withholding cooperation) players who have previously defected on others, reward punishers by cooperating with players who have punished selfish players, and reward or punish those who do or do not punish nonpunishers (17, 87). Second, by developing a utility-based model, our framework is sensitive to the payoffs and structure of the game itself. This allows for generalization beyond the Game Generator to extended interactions across space and time such as video games, human–algorithm interactions, or even human–robot interactions (49, 51, 88–90). Last, unlike previous reputation systems such as the leading eight (14), the Bayesian Reciprocator operates with graded evaluations: the more observations of a player cooperating or defecting on others, the more evidence the player has that the player is a cooperator (or altruistic) or is selfish (and should be punished). An emergent feature of this gradedness is that as the Bayesian Reciprocator is more sure it is interacting with another Bayesian Reciprocator (beliefs closer to 1), it is willing to pay a higher relative price for a collective benefit (26, 91).

Compared to prior approaches, the gradedness of the Bayesian Reciprocator is key to its robustness to execution errors. While the cooperative automata strategies fully break down once the error rate exceeds 0.1, the Bayesian Reciprocator still outcompetes all others at 2 to 3 times that error rate. This works because the Bayesian Reciprocator reasons probabilistically about errors and treats them as a standard statistical learning problem where evidence is accumulated over many time steps. The extent that a noncooperative action should be treated as the player’s true intention versus explained away as an error is calibrated automatically by a probabilistic update. Consequently, when the Bayesian Reciprocator becomes more and more sure (belief closer to 1) that another player is of the same type, they will also be more likely to forgive errors. Similar to how humans treat the importance of first impressions, the Bayesian Reciprocator is less likely to forgive errors early on than later on when beliefs are already converged. This type of commonsense social reasoning is not present in any of the forgiveness mechanisms of the behavioral automata. GTFT forgives defection with a fixed probability, Forgiver always forgives, and WSLS forgives through an error correction mechanism that also makes it less successful at resisting defectors (WSLS cooperates 50% of the time with AllD).

We are not the first to consider the evolution of utility-based preferences for cooperation. However, prior works required that utility functions are publicly observable (92) or assortment was required in the matching process to reach a cooperative equilibrium (42). These prior models did not require inferential machinery or develop mechanisms for reciprocity. Finally, compared to other type-based cooperators, there is no need to invent a signaling system that enables similarity-based conditional cooperation such as tags or “green-beards” (93–95). For the Bayesian Reciprocator, the utility function is both a signal for the conditional cooperation of others and the causal determinant of a player’s behavior. As such, the behavior of the Bayesian Reciprocator is a signal of conditional cooperation that cannot be faked or imitated without adopting it. The Bayesian Reciprocator could leverage tags or other public features that are diagnostic for type to speed up cooperation by setting the initial prior. By only affecting the prior, a false signaler that “looks like” a cooperator would be quickly detected as a cheater after defecting just a few times.

Future work can leverage the modeling framework we have introduced here to study many key features of human cooperation. For instance, explicit forms of punishment, where a player pays a cost to reduce the payoffs of others as retribution or as a teaching signal (96, 97), could be modeled by changing the sign of the other player’s payoffs in Eq. **2**. Other relevant features for structuring cooperation, such as fairness, partner choice, or rules, could also be considered in our framework by extending the Game Generator and modifying the utility function (98–101). While in this work, we studied players with a fixed prior, the prior could be learned hierarchically both during interactions with multiple partners or inherited culturally across generations (29, 61). Sophisticated structure learners could learn and pass on not just the weighting over types but even discover the types themselves. Nonparametric Bayesian inference (102) or program learning (103) could be used to implicitly represent infinite player types. These flexible priors allow the sophistication of represented types to grow dynamically with the complexity of the data.

While the Bayesian Reciprocator represents only one account of how human-like cooperation could operate and arise stably, its principles, mathematical foundations, and computational structures could be generally useful in building more cooperative AI. Ideally, an AI that operates in the human world will have a human-like theory of mind that can be leveraged to understand, learn from, and cooperate with people. As AI is increasingly making and advising on decisions in areas ranging from autonomous cars to public policy, these systems will confront many of the same social challenges studied here: recognizing the cooperative intent (or lack thereof) of others, inferring reputation from interaction and observation, reciprocating proportionately, and more (90, 104, 105). These issues arise when AI systems need to understand the dynamics of cooperation between human agents, as well as in new forms of cooperation that could emerge or be engineered in human–AI interactions or AI–AI interactions. In each case, agents may not share the same goals and will need to both reason about each others’ intentions and figure out whom to cooperate with in order to achieve mutual benefit. More broadly, the capacity to reason about human intentions and utility functions may be essential in aligning AI systems with human values (106, 107). Our analysis of recursive and adaptively weighted utility functions and Bayesian theory of mind inferences underlying general patterns of cooperation could be a central part of this alignment landscape.

They hypothesized that a player with theory of mind could resist extortion and other manipulations and ultimately conclude, “it is exactly evolution, on the hugely larger canvas of DNA-based life, that ultimately has produced X, the player with the mind.” In this work, we realize this hypothesis by developing the Bayesian Reciprocator, a model for the evolution of cooperation that leverages theory of mind for a distinct cooperative advantage. Quantifying this advantage in evolutionary game theoretic terms shows why humans, the most sophisticated cooperators, also have the most sophisticated machinery for understanding the minds of others.

## Materials and Methods

### Bayesian Reciprocator.

In Algorithm 1, we show the pseudocode for the Bayesian Reciprocator belief updates. The core belief update happens on lines 22 to 30. On line 28, internal models of other player types are updated with the latest observation. For example, if m=TFT, the state of the *TFT* automata will be updated with the latest action. The simulations utilize additional optimizations that have been omitted for clarity but are present in the source code. We take advantage of the fact that observer subsets form a partially ordered set. This allows the Bayesian Reciprocator to initialize the observer subsets only when they occur in the game and initialize their beliefs from the next larger subset when it is available. These optimizations reduce computation and memory consumption when simulating a population of players.

When interactions are private, the number of observer subsets scales linearly with the number of players because the Bayesian Reciprocator must store the beliefs that each pair of players have about each other. When all interactions are fully observable, they still scale linearly since the Bayesian Reciprocator only needs a single observer subset that corresponds to the joint beliefs held by all. When observations are partially observable, the Bayesian Reciprocator must track each unique observer subset that occurs. In the worst case, every unique combination occurs. This worst case would require representing a powerset of the observers and require $2^{N}$ observer subsets.

### Game Generator.

The generative process for the Game Generator is defined as follows. To create a sample *G*_*i*_, we first sample the number of players (including the decision maker) and the number of choice types (including “do nothing”). The number of players is 2 or 3 with equal probability in the evolutionary analyses and 2 in the intragenerational learning experiments shown for the intragenerational studied shown in Figs. 4*A* and 5*A*.

The number of choice types is Poisson(2). For each choice type, we sample a cost c∼Poisson(C) so that costs are sometimes zero and a benefit b∼Exponential(B). For each *c*, *b*, and non-decision-making player, we create a choice option where the decision-maker pays *c* to give the non-decision-making player *c* + *b*. This ensures that the benefit is always larger than the cost. Finally, a do nothing choice option is added where all payoffs are 0. Each interaction has a unique probability of a “trembling hand” where with probability *ϵ*, a different action is taken instead of the action chosen. Each interaction is observed by all players with probability *ω* and is otherwise only observed by those interacting in *G*_*i*_. Repeated interactions are generated by having the same set of players sample a new *G*_*i*_ after each decision.

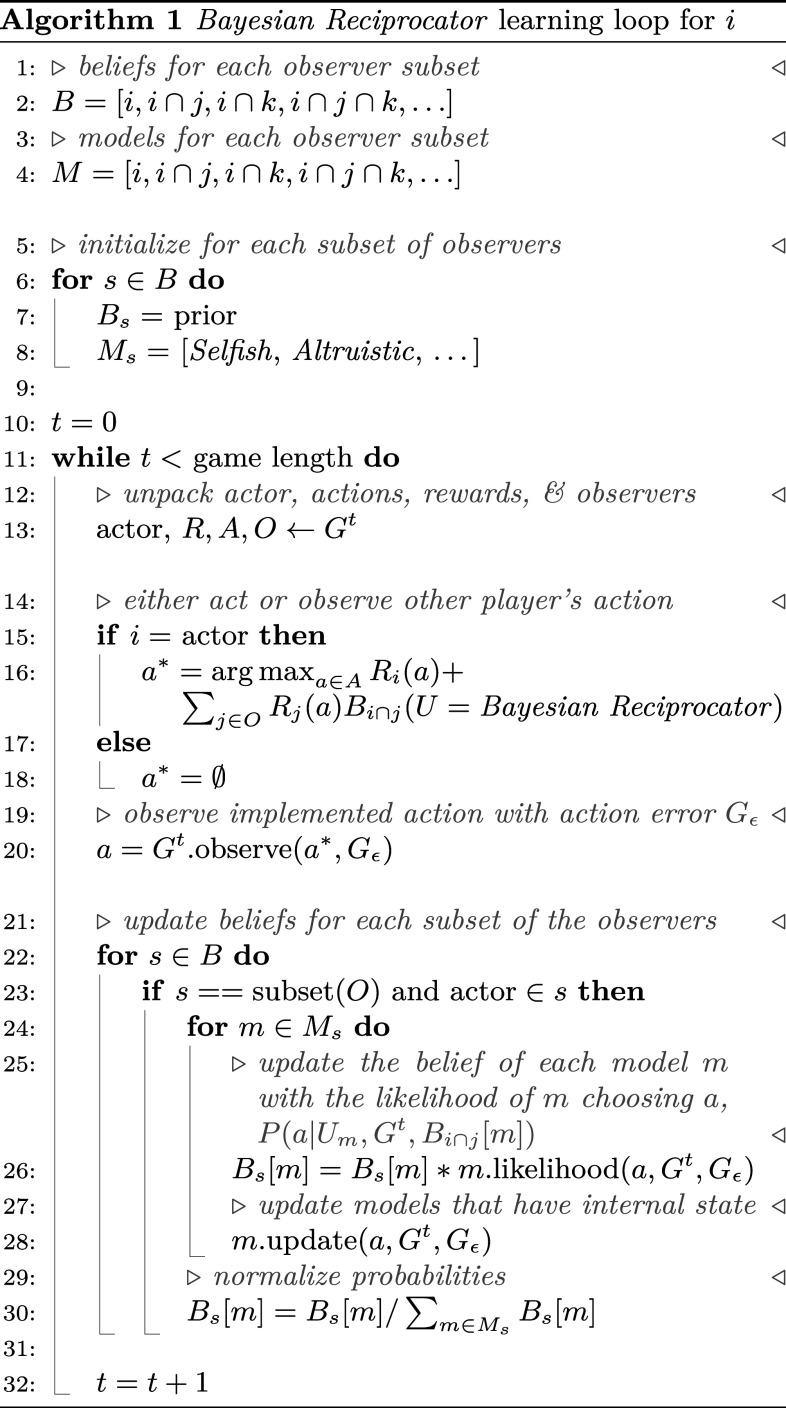

### Automata Strategies.

The memory-1 automata strategies can be defined by a vector for four numbers: $\left(p_{\mathrm{cc}},p_{\mathrm{cd}},p_{\mathrm{dc}},p_{\mathrm{dd}}\right)$, i.e., the probability of cooperating after both players cooperated, the player cooperated and the opponent defected, player defected, and the opponent cooperated, the player defected and the opponent defected. AllD is (0,0,0,0), AllC is (1,1,1,1), TFT is (1,0,1,0), WSLS is (1,0,0,1), GTFT is (1,0.66,1,0.66), Forgiver is (1,0,1,1), and Extort2 is (0.85,0.5,0.35,0) (7, 8, 24, 83). The specific values for GTFT were computed as optimal for IPD with *B* = 3 and *C* = 1 (7). Extort2 is an extortion strategy with *χ* = 2 (83). AllC, TFT, WSLS, GTFT, and Forgiver play cooperate on the first round. AllD and Extort2 play defect on the first round.

### Evolutionary Analysis of the Game Generator.

We simulate an evolutionary selection process to compute the steady-state abundance of each strategy at equilibrium using the finite population Moran Process. In the Moran process, in each generation, one player is chosen at random, and that player chooses another player (inclusive of itself) with probability proportional to its cumulative payoff and then either copies that player’s type or mutates into a random player type with probability *δ* (4, 75).

The evolutionary analysis of the Game Generator was done in a population of *N* = 10 players with mutation rate *δ* = 0.001 and selection strength *s* = 2. Results were robust to the choice of these parameters. We first calculate the expected cumulative payoff to each type for each composition of player types. So if there are *M* = 3 player types (Bayesian Reciprocator, Selfish, Altruistic) in a population of 10, we compute the expected cumulative payoffs for each of the population compositions (9,1,0),(8,2,0),…,(1,0,9), where the number in each position is the number of players of that type in the population. Expected payoffs were calculated empirically by averaging over 200 simulations.

For each composition, we take a softmax of the expected cumulative payoffs controlled by the selection strength (s) to get the probability that each player type will be copied (109). For a composition *i* out of *C* total compositions, the probability of choosing type *t* is:

$$\begin{array}{c} \mu_{t}^{i}=\frac{e^{s\times \pi_{t}^{i}}}{\sum_{m=0}^{M}e^{s\times \pi_{m}^{i}}}, \end{array}$$

where $\pi_{t}^{i}$ is the expected cumulative payoff of type *t* in composition *i*. Expected cumulative payoffs were calculated empirically by averaging over 200 simulations. Finally, *p* is a *C* × *C* transition matrix where each element $p_{i,j}$ is the probability of transitioning from population composition *i* to population composition *j* in a single time step. Let *b* be the player type that increased from i→j and let *d* be the player type that decreased from i→j. Then:

$$\begin{array}{c} p_{i,j}=\left(\delta \left(1/M\right)+\left(1-\delta \right)\mu_{b}^{i}\right)\left(\;{\text{pop}}_{d}/N\right), \end{array}$$

where $\mu_{b}^{i}$ is the probability of choosing to copy *b*, the player type that increased when moving from *i* to *j* and $\;{\text{pop}}_{d}^{i}$ is the population size of type *d*, the player type that decreased when moving from *i* to *j*. The first term is the probability that type *b* increased due to mutation, the second term is the probability that type *b* increased due to selection, and the third term is the probability that type *d* was randomly chosen to change strategies.

Thus, *p* is a transition matrix that defines a stochastic process between possible populations of player types. Let $x_{i}^{*}$ be the steady state frequency of population composition *i*. To find the steady state of this process, we compute a distribution of frequencies that satisfies:

$$\begin{array}{c} \left[\begin{array}{c} x_{1}^{*}\\ \vdots \\ x_{C}^{*} \end{array}\right]=\left[\begin{array}{ccc} 1-\sum_{i}p_{i,1} & \cdots & p_{1,C}\\ \vdots & \ddots & \vdots \\ p_{C,1} & \cdots & 1-\sum_{i}p_{i,C} \end{array}\right]\left[\begin{array}{c} x_{1}^{*}\\ \vdots \\ x_{C}^{*} \end{array}\right]. \end{array}$$

The steady state $x^{*}$ is the eigenvector corresponding to the largest eigenvalue of matrix *p*. The frequency of player type *t* in steady state is computed by summing over the population compositions: $\frac{1}{N}\sum_{i}i_{t}\times x_{i}^{*}$, where *i*_*t*_ is the number of players of type *t* in population *i*. See ref. 4 for a detailed mathematical treatment of this stochastic process.

### Evolutionary Analysis of the IPD.

The evolutionary analysis of the IPD was done in the low mutation limit (δ→0) in a population of *N* = 100 players and selection strength *s* = 1 following the calculations of refs. 109 and 110. In the low mutation limit, the population spends most of its time in a homogenous state with only one player type. A new player type either takes over the entire population (fixation) or dies out. This allows us to calculate the transition probability between pairs of strategy types rather than all combinatorial combinations. We calculate the steady state distribution of *M* player types by constructing an *M* × *M* transition matrix, *p*, between homogenous populations of player types from the expected payoffs of each pair of player types. Then $p_{i,j}$ is the probability of transitioning from a homogenous population of type *i* to a homogenous population of type *j*.

$$\begin{array}{c} p_{i,j}=\frac{1}{1+\sum_{n=1}^{N-1}\prod_{k=1}^{n}\mu_{i}^{k}/\mu_{j}^{k}}, \end{array}$$

where $\mu_{i}^{k}$ is the exponentiated payoffs of player types *i* and *j* in a population composition of *ki*-players and *N* − *kj*-players:

$$\begin{array}{c} \mu_{i}^{k}=\;\exp\left(s*(\frac{k-1}{N-1}\pi_{i,i}+\frac{N-k-1}{N-1}\pi_{i,j})\right) \end{array}$$

and $\pi_{i,i}$ are the expected payoff of player type *i* paired with itself and $\pi_{i,j}$ is the expected payoff of player type *i* paired with player type *j*. Expected payoffs were calculated empirically by averaging over 1,000 simulations.

Thus, *p* is a transition matrix that defines a stochastic process between types of players. Let $x_{i}^{*}$ be the steady state frequency of type *i*. To find the steady state of this process, we compute a distribution of frequencies that satisfies:

$$\begin{array}{c} \left[\begin{array}{c} x_{1}^{*}\\ \vdots \\ x_{M}^{*} \end{array}\right]=\left[\begin{array}{ccc} 1-\sum_{i}p_{i,1} & \cdots & p_{1,M}\\ \vdots & \ddots & \vdots \\ p_{M,1} & \cdots & 1-\sum_{i}p_{i,M} \end{array}\right]\left[\begin{array}{c} x_{1}^{*}\\ \vdots \\ x_{M}^{*} \end{array}\right]. \end{array}$$

The steady state $x^{*}$ is the eigenvector corresponding to the largest eigenvalue of matrix *p*.

## Supplementary Material

Appendix 01 (PDF)

## Data Availability

Source code has been deposited in GitHub (https://github.com/maxkw/evolution) (111).

## References

[1] R. L. Trivers, The evolution of reciprocal altruism. Q. R. Biol. **46**, 35–57 (1971).

[2] R. Axelrod, W. D. Hamilton, The evolution of cooperation. Science **211**, 1390–1396 (1981). 7466396 10.1126/science.7466396

[3] M. A. Nowak, Five rules for the evolution of cooperation. Science **314**, 1560–1563 (2006). 17158317 10.1126/science.1133755 PMC3279745

[4] K. Sigmund, The Calculus of Selfishness (Princeton University Press, 2010).

[5] D. G. Rand, M. A. Nowak, Human cooperation. Trends Cogn. Sci. **17**, 413 (2013). 23856025 10.1016/j.tics.2013.06.003

[6] R. Boyd, A Different Kind of Animal: How Culture Transformed Our Species (Princeton University Press, 2017), vol. 46.

[7] M. A. Nowak, K. Sigmund, Tit for tat in heterogenous populations. Nature **355**, 250 (1992).

[8] M. Nowak, K. Sigmund, A strategy of win-stay, lose-shift that outperforms tit-for-tat in the prisoner’s dilemma game. Nature **364**, 56 (1993). 8316296 10.1038/364056a0

[9] C. Hilbe, L. A. Martinez-Vaquero, K. Chatterjee, M. A. Nowak, Memory-n strategies of direct reciprocity. Proc. Natl. Acad. Sci. U.S.A. **114**, 4715–4720 (2017). 28420786 10.1073/pnas.1621239114 PMC5422766

[10] C. Hilbe, K. Chatterjee, M. A. Nowak, Partners and rivals in direct reciprocity. Nat. Hum. Behav. **2**, 469–477 (2018). 31097794 10.1038/s41562-018-0320-9

[11] M. A. Nowak, K. Sigmund, Evolution of indirect reciprocity by image scoring. Nature **393**, 573–577 (1998). 9634232 10.1038/31225

[12] K. Panchanathan, R. Boyd, Indirect reciprocity can stabilize cooperation without the second-order free rider problem. Nature **432**, 499–502 (2004). 15565153 10.1038/nature02978

[13] M. A. Nowak, K. Sigmund, Evolution of indirect reciprocity. Nature **437**, 1291–1298 (2005). 16251955 10.1038/nature04131

[14] H. Ohtsuki, Y. Iwasa, The leading eight: Social norms that can maintain cooperation by indirect reciprocity. J. Theor. Biol. **239**, 435–444 (2006). 16174521 10.1016/j.jtbi.2005.08.008

[15] C. Hilbe, L. Schmid, J. Tkadlec, K. Chatterjee, M. A. Nowak, Indirect reciprocity with private, noisy, and incomplete information. Proc. Natl. Acad. Sci. U.S.A. **115**, 12241–12246 (2018). 30429320 10.1073/pnas.1810565115 PMC6275544

[16] L. Schmid, K. Chatterjee, C. Hilbe, M. A. Nowak, A unified framework of direct and indirect reciprocity. Nat. Hum. Behav. **5**, 1292–1302 (2021). 33986519 10.1038/s41562-021-01114-8

[17] J. J. Jordan, A pull versus push framework for reputation. Trends Cogn. Sci. **27**, 852–866 (2023). 37468335 10.1016/j.tics.2023.06.005

[18] J. Bednar, S. Page, Can game(s) theory explain culture? The emergence of cultural behavior within multiple games. Ration. Soc. **19**, 65–97 (2007).

[19] C. Hilbe, Š. Šimsa, K. Chatterjee, M. A. Nowak, Evolution of cooperation in stochastic games. Nature **559**, 246–249 (2018). 29973718 10.1038/s41586-018-0277-x

[20] Q. Su, A. McAvoy, L. Wang, M. A. Nowak, Evolutionary dynamics with game transitions. Proc. Natl. Acad. Sci. U.S.A. **116**, 25398–25404 (2019). 31772008 10.1073/pnas.1908936116 PMC6926053

[21] W. Qi, E. Vul, The evolution of theory of mind on welfare tradeoff ratios. Evol. Hum. Behav. **43**, 381–393 (2022).

[22] M. A. Nowak, K. Sigmund, The alternating prisoner’s dilemma. J. Theor. Biol. **168**, 219–226 (1994).

[23] M. R. Frean, The prisoner’s dilemma without synchrony. Proc. R. Soc. Lond. B, Biol. Sci. **257**, 75–79 (1994). 10.1098/rspb.1994.0096 8090793

[24] B. M. Zagorsky, J. G. Reiter, K. Chatterjee, M. A. Nowak, Forgiver triumphs in alternating prisoner’s dilemma. PLoS ONE **8**, e80814 (2013). 24349017 10.1371/journal.pone.0080814 PMC3861238

[25] P. S. Park, M. A. Nowak, C. Hilbe, Cooperation in alternating interactions with memory constraints. Nat. Commun. **13**, 1–11 (2022). 35136025 10.1038/s41467-022-28336-2 PMC8825791

[26] G. Roberts, T. N. Sherratt, Development of cooperative relationships through increasing investment. Nature **394**, 175–179 (1998). 9671299 10.1038/28160

[27] A. McAvoy, C. Hauert, Autocratic strategies for iterated games with arbitrary action spaces. Proc. Natl. Acad. Sci. U.S.A. **113**, 3573–3578 (2016). 26976578 10.1073/pnas.1520163113 PMC4822593

[28] S. Pinker, The cognitive niche: Coevolution of intelligence, sociality, and language. Proc. Natl. Acad. Sci. U.S.A. **107**, 8993–8999 (2010). 20445094 10.1073/pnas.0914630107 PMC3024014

[29] A. Peysakhovich, D. G. Rand, Habits of virtue: Creating norms of cooperation and defection in the laboratory. Manag. Sci. **62**, 631–647 (2015).

[30] D. G. Rand , Social heuristics shape intuitive cooperation. Nat. Commun. **5**, 3677 (2014). 24751464 10.1038/ncomms4677

[31] F. Heider, Attitudes and cognitive organization. J. Psychol. **21**, 107–112 (1946). 21010780 10.1080/00223980.1946.9917275

[32] J. M. van Baar, M. R. Nassar, W. Deng, O. FeldmanHall, Latent motives guide structure learning during adaptive social choice. Nat. Hum. Behav. **6**, 404–414 (2022). 34750584 10.1038/s41562-021-01207-4 PMC13285670

[33] J. Heffner, O. FeldmanHall, A probabilistic map of emotional experiences during competitive social interactions. Nat. Commun. **13**, 1718 (2022). 35361768 10.1038/s41467-022-29372-8 PMC8971394

[34] D. Premack, G. Woodruff, Does the chimpanzee have a theory of mind? Behav. Brain Sci. **1**, 515–526 (1978).

[35] H. M. Wellman, *The Child’s Theory of Mind* (The MIT Press, Cambridge, MA, 1992).

[36] S. Liu, T. D. Ullman, J. B. Tenenbaum, E. S. Spelke, Ten-month-old infants infer the value of goals from the costs of actions. Science **358**, 1038–1041 (2017). 29170232 10.1126/science.aag2132

[37] J. Kiley Hamlin, T. Ullman, J. Tenenbaum, N. Goodman, C. Baker, The mentalistic basis of core social cognition: Experiments in preverbal infants and a computational model. Dev. Sci. **16**, 209–226 (2013). 23432831 10.1111/desc.12017 PMC4100482

[38] F. Ting, R. Baillargeon, Toddlers draw broad negative inferences from wrongdoers’ moral violations. Proc. Natl. Acad. Sci. U.S.A. **118**, e2109045118 (2021). 34544874 10.1073/pnas.2109045118 PMC8488678

[39] J. Jara-Ettinger, H. Gweon, J. B. Tenenbaum, L. E. Schulz, Children’s understanding of the costs and rewards underlying rational action. Cognition **140**, 14–23 (2015). 25867996 10.1016/j.cognition.2015.03.006

[40] R. S. Sutton, A. G. Barto, Reinforcement Learning: An Introduction (MIT Press, 2018).

[41] E. Dekel, J. C. Ely, O. Yilankaya, Evolution of preferences. Rev. Econ. Stud. **74**, 685–704 (2007).

[42] I. Alger, J. W. Weibull, Homo moralis–preference evolution under incomplete information and assortative matching. Econometrica **81**, 2269–2302 (2013).

[43] M. Kleiman-Weiner, R. Saxe, J. B. Tenenbaum, Learning a commonsense moral theory. Cognition **167**, 107–123 (2017). 28351662 10.1016/j.cognition.2017.03.005

[44] M. Rabin, Incorporating fairness into game theory and economics. Am. Econ. Rev. **83**, 1281–1302 (1993).

[45] M. Dufwenberg, G. Kirchsteiger, A theory of sequential reciprocity. Games Econom. Behav. **47**, 268–298 (2004).

[46] A. Falk, U. Fischbacher, A theory of reciprocity. Games Econom. Behav. **54**, 293–315 (2006).

[47] W. Yoshida, R. J. Dolan, K. J. Friston, Game theory of mind. PLoS Comput. Biol. **4**, e1000254 (2008). 19112488 10.1371/journal.pcbi.1000254 PMC2596313

[48] T. Ullman , Help or hinder: Bayesian models of social goal inference in Adv. Neural Inf. Process. Syst., 1874–1882 (2009).

[49] M. Kleiman-Weiner, M. K. Ho, J. L. Austerweil, M. L. Littman, J. B. Tenenbaum, “Coordinate to cooperate or compete: Abstract goals and joint intentions in social interaction” in *Proceedings of the 38th Annual Conference of the Cognitive Science Society* (2016).

[50] K. Khalvati , Modeling other minds: Bayesian inference explains human choices in group decision-making. Sci. Adv. **5**, eaax8783 (2019). 31807706 10.1126/sciadv.aax8783 PMC6881156

[51] M. Shum, M. Kleiman-Weiner, M. L. Littman, J. B. Tenenbaum, “Theory of minds: Understanding behavior in groups through inverse planning” in *Proceedings of the AAAI Conference on Artificial Intelligence* (2019), vol. 33, pp. 6163–6170.

[52] D. A. Pizarro, D. Tannenbaum, “Bringing character back: How the motivation to evaluate character influences judgments of moral blame” in *The Social Psychology of Morality: Exploring the Causes of Good and Evil* (2011), M. Mikulincer, P. R. Shaver, Eds. (American Psychological Association, Washington, DC, 2011), pp. 91–108.

[53] E. L. Uhlmann, D. A. Pizarro, D. Diermeier, A person-centered approach to moral judgment. Perspect. Psychol. Sci. **10**, 72–81 (2015). 25910382 10.1177/1745691614556679

[54] J. Wattles, *The Golden Rule* (Oxford University Press, UK, 1997).

[55] O. Weisel, S. Shalvi, The collaborative roots of corruption. Proc. Natl. Acad. Sci. U.S.A. **112**, 10651–10656 (2015). 26261341 10.1073/pnas.1423035112 PMC4553769

[56] C. L. Baker, J. Jara-Ettinger, R. Saxe, J. B. Tenenbaum, Rational quantitative attribution of beliefs, desires and percepts in human mentalizing. Nat. Hum. Behav. **1**, 0064 (2017).

[57] R. D. Hawkins , From partners to populations: A hierarchical Bayesian account of coordination and convention. Psychol. Rev. **130**, 977 (2023). 35420850 10.1037/rev0000348

[58] S. V. Albrecht, P. Stone, Autonomous agents modelling other agents: A comprehensive survey and open problems. Artif. Intell. **258**, 66–95 (2018).

[59] M. Ramırez, H. Geffner, “Goal recognition over POMDPs: Inferring the intention of a POMDP agent in IJCAI” in *IJCAI/AAAI, 2009–2014* (2011).

[60] J. Jara-Ettinger, Theory of mind as inverse reinforcement learning. Curr. Opin. Behav. Sci. **29**, 105–110 (2019).

[61] C. Kemp, A. Perfors, J. B. Tenenbaum, Learning overhypotheses with hierarchical Bayesian models. Dev. Sci. **10**, 307–321 (2007). 17444972 10.1111/j.1467-7687.2007.00585.x

[62] B. Liddle, D. Nettle, Higher-order theory of mind and social competence in school-age children. J. Cult. Evol. Psychol. **4**, 231–244 (2006).

[63] G. Fu, W. S. Xiao, M. Killen, K. Lee, Moral judgment and its relation to second-order theory of mind. Dev. Psychol. **50**, 2085 (2014). 24866286 10.1037/a0037077

[64] P. J. Gmytrasiewicz, P. Doshi, A framework for sequential planning in multi-agent settings. J. Artif. Intell. Res. **24**, 49–79 (2005).

[65] M. Costa-Gomes, V. P. Crawford, B. Broseta, Cognition and behavior in normal-form games: An experimental study. Econometrica, 1193–1235 (2001).

[66] C. F. Camerer, T. H. Ho, J. K. Chong, A cognitive hierarchy model of games. Q. J. Econ., 861–898 (2004).

[67] J. R. Wright, K. Leyton-Brown, “Beyond equilibrium: Predicting human behavior in normal-form games” in *AAAI* (2010).

[68] L. Zettlemoyer, B. Milch, L. Kaelbling, Multi-agent filtering with infinitely nested beliefs. Adv. Neural Inf. Process. Syst. **21** (2008).

[69] J. Y. Halpern, M. Y. Vardi, “Model checking vs. theorem proving: A manifesto” in *Artificial Intelligence and Mathematical Theory of Computation*, V. Lifschitz, Ed. (Academic Press, 1991), pp. 151–176.

[70] R. Fagin, J. Y. Halpern, Reasoning about knowledge and probability. J. ACM **41**, 340–367 (1994).

[71] R. Fagin, J. Y. Halpern, Y. Moses, M. Vardi, Reasoning About Knowledge (MIT Press, 2004).

[72] K. A. Thomas, P. DeScioli, O. S. Haque, S. Pinker, The psychology of coordination and common knowledge. J. Pers. Soc. Psychol. **107**, 657 (2014). 25111301 10.1037/a0037037

[73] J. De Freitas, K. Thomas, P. DeScioli, S. Pinker, Common knowledge, coordination, and strategic mentalizing in human social life. Proc. Natl. Acad. Sci. U.S.A. **116**, 13751–13758 (2019). 31253709 10.1073/pnas.1905518116 PMC6628641

[74] T. Nagel, The View from Nowhere (Oxford University Press, 1986).

[75] M. A. Nowak, A. Sasaki, C. Taylor, D. Fudenberg, Emergence of cooperation and evolutionary stability in finite populations. Nature **428**, 646–650 (2004). 15071593 10.1038/nature02414

[76] D. Fudenberg, L. A. Imhof, Imitation processes with small mutations. J. Econ. Theory **131**, 251–262 (2006).

[77] J. García, M. van Veelen, In and out of equilibrium. I: Evolution of strategies in repeated games with discounting. J. Econ. Theory **161**, 161–189 (2016).

[78] L. A. Imhof, D. Fudenberg, M. A. Nowak, Evolutionary cycles of cooperation and defection. Proc. Natl. Acad. Sci. U.S.A. **102**, 10797–10800 (2005). 16043717 10.1073/pnas.0502589102 PMC1182423

[79] J. García, M. Van Veelen, No strategy can win in the repeated prisoner’s dilemma: Linking game theory and computer simulations. Front. Rob. AI **5**, 102 (2018). 10.3389/frobt.2018.00102 PMC7805755 33500981

[80] E. Fehr, K. M. Schmidt, A theory of fairness, competition, and cooperation. Q. J. Econ. **114**, 817–868 (1999).

[81] Y. Fujimoto, H. Ohtsuki, Evolutionary stability of cooperation in indirect reciprocity under noisy and private assessment. Proc. Natl. Acad. Sci. U.S.A. **120**, e2300544120 (2023). 37155910 10.1073/pnas.2300544120 PMC10194006

[82] R. Axelrod, The Evolution of Cooperation (Basic Books, 1985).

[83] W. H. Press, F. J. Dyson, Iterated prisoner’s dilemma contains strategies that dominate any evolutionary opponent. Proc. Natl. Acad. Sci. U.S.A. **109**, 10409–10413 (2012). 22615375 10.1073/pnas.1206569109 PMC3387070

[84] A. J. Stewart, J. B. Plotkin, From extortion to generosity, evolution in the iterated prisoner’s dilemma. Proc. Natl. Acad. Sci. U.S.A. **110**, 15348–15353 (2013). 24003115 10.1073/pnas.1306246110 PMC3780848

[85] R. Boyd, P. J. Richerson, J. Henrich, The cultural niche: Why social learning is essential for human adaptation. Proc. Natl. Acad. Sci. U.S.A. **108**, 10918–10925 (2011). 21690340 10.1073/pnas.1100290108 PMC3131818

[86] M. Kleiman-Weiner , “Downloading culture. Zip: Social learning by program induction” in *42nd Annual Meeting of the Cognitive Science Society: Developing a Mind: Learning in Humans, Animals, and Machines CogSci* (2020).

[87] J. W. Martin, J. J. Jordan, D. G. Rand, F. Cushman, When do we punish people who don’t? Cognition **193**, 104040 (2019). 31408816 10.1016/j.cognition.2019.104040

[88] M. L. Littman, Markov games as a framework for multi-agent reinforcement learning. ICML **94**, 157–163 (1994).

[89] J. Z. Leibo, V. Zambaldi, M. Lanctot, J. Marecki, T. Graepel, “Multi-agent reinforcement learning in sequential social dilemmas” in *Proceedings of the 16th Conference on Autonomous Agents and MultiAgent Systems* (International Foundation for Autonomous Agents and Multiagent Systems, 2017), pp. 464–473.

[90] J. W. Crandall , Cooperating with machines. Nat. Commun. **9**, 233 (2018). 29339817 10.1038/s41467-017-02597-8 PMC5770455

[91] M. Schmelz, S. Grueneisen, A. Kabalak, J. Jost, M. Tomasello, Chimpanzees return favors at a personal cost. Proc. Natl. Acad. Sci. U.S.A. **114**, 7462–7467 (2017). 28630319 10.1073/pnas.1700351114 PMC5514715

[92] T. C. Bergstrom, On the evolution of altruistic ethical rules for siblings. Am. Econ. Rev. **85**, 58–81 (1995).

[93] R. Dawkins, N. Davis, The Selfish Gene (Macat Library, 2017).

[94] R. L. Riolo, M. D. Cohen, R. Axelrod, Evolution of cooperation without reciprocity. Nature **414**, 441–443 (2001). 11719803 10.1038/35106555

[95] V. A. Jansen, M. Van Baalen, Altruism through beard chromodynamics. Nature **440**, 663–666 (2006). 16572169 10.1038/nature04387

[96] R. Boyd, H. Gintis, S. Bowles, P. J. Richerson, The evolution of altruistic punishment. Proc. Natl. Acad. Sci. U.S.A. **100**, 3531–3535 (2003). 12631700 10.1073/pnas.0630443100 PMC152327

[97] A. Sarin, M. K. Ho, J. W. Martin, F. A. Cushman, Punishment is organized around principles of communicative inference. Cognition **208**, 104544 (2021). 33383397 10.1016/j.cognition.2020.104544

[98] N. Baumard, J. B. André, D. Sperber, A mutualistic approach to morality: The evolution of fairness by partner choice. Behav. Brain Sci. **36**, 59–78 (2013). 23445574 10.1017/S0140525X11002202

[99] D. G. Rand, C. E. Tarnita, H. Ohtsuki, M. A. Nowak, Evolution of fairness in the one-shot anonymous ultimatum game. Proc. Natl. Acad. Sci. U.S.A. **110**, 2581–2586 (2013). 23341593 10.1073/pnas.1214167110 PMC3574936

[100] S. Levine, M. Kleiman-Weiner, L. Schulz, J. Tenenbaum, F. Cushman, The logic of universalization guides moral judgment. Proc. Natl. Acad. Sci. U.S.A. **117**, 26158–26169 (2020). 33008885 10.1073/pnas.2014505117 PMC7584905

[101] J. Li , Evolution of cooperation through cumulative reciprocity. Nat. Comput. Sci. **2**, 677–686 (2022). 38177263 10.1038/s43588-022-00334-w

[102] M. Kleiman-Weiner, J. B. Tenenbaum, P. Zhou, Non-parametric Bayesian inference of strategies in infinitely repeated games. Econometrics J. **21**, 298–315 (2017).

[103] K. Ellis , Dreamcoder: Growing generalizable, interpretable knowledge with wake-sleep Bayesian program learning. Philos. Trans. R. Soc. A **381**, 20220050 (2023). 10.1098/rsta.2022.0050 37271169

[104] J. F. Bonnefon, A. Shariff, I. Rahwan, The social dilemma of autonomous vehicles. Science **352**, 1573–1576 (2016). 27339987 10.1126/science.aaf2654

[105] A. Dafoe , Open problems in cooperative AI. arXiv [Preprint] (2020). https://arxiv.org/abs/2012.08630 (Accessed 15 December 2020).

[106] D. Hadfield-Menell, S. J. Russell, P. Abbeel, A. Dragan, Cooperative inverse reinforcement learning. Adv. Neural Inf. Process. Syst. **29**, 3909–3917 (2016).

[107] S. Russell, Human Compatible: Artificial Intelligence and the Problem of Control (Penguin, 2019).

[108] C. Hilbe, A. Traulsen, K. Sigmund, Partners or rivals? Strategies for the iterated prisoner’s dilemma. Games Econom. Behav. **92**, 41–52 (2015). 10.1016/j.geb.2015.05.005 PMC4547490 26339123

[109] D. G. Rand, M. A. Nowak, The evolution of antisocial punishment in optional public goods games. Nat. Commun. **2**, 434 (2011). 21847108 10.1038/ncomms1442 PMC3279747

[110] A. Bear, D. G. Rand, Intuition, deliberation, and the evolution of cooperation. Proc. Natl. Acad. Sci. U.S.A. **113**, 936–941 (2016). 26755603 10.1073/pnas.1517780113 PMC4743833

[111] M. Kleiman-Weiner, A. Vientós, D. G. Rand, J. B. Tenenbaum, maxkw/evolution. Github. https://github.com/maxkw/evolution. Deposited 18 March 2025.

