# Supplementary material for: Evolving general cooperation with a Bayesian theory of mind
Source: Proc Natl Acad Sci U S A. 2025 Jun 16;122(25):e2400993122. doi: 10.1073/pnas.2400993122 (PMC12207496; doi:10.1073/pnas.2400993122)
Supplement: Supplementary file 1 — Appendix 01 (PDF) [file pnas.2400993122.sapp.pdf]

PNAS

Supporting Information for

Evolving General Cooperation with a Bayesian Theory of Mind

Max Kleiman-Weiner, Alejandro Vientós, David G. Rand and Joshua B. Tenenbaum

Corresponding Author: Max Kleiman-Weiner
E-mail: maxkw@uw.edu

This PDF file includes:

Figs. S1 to S6
SI References

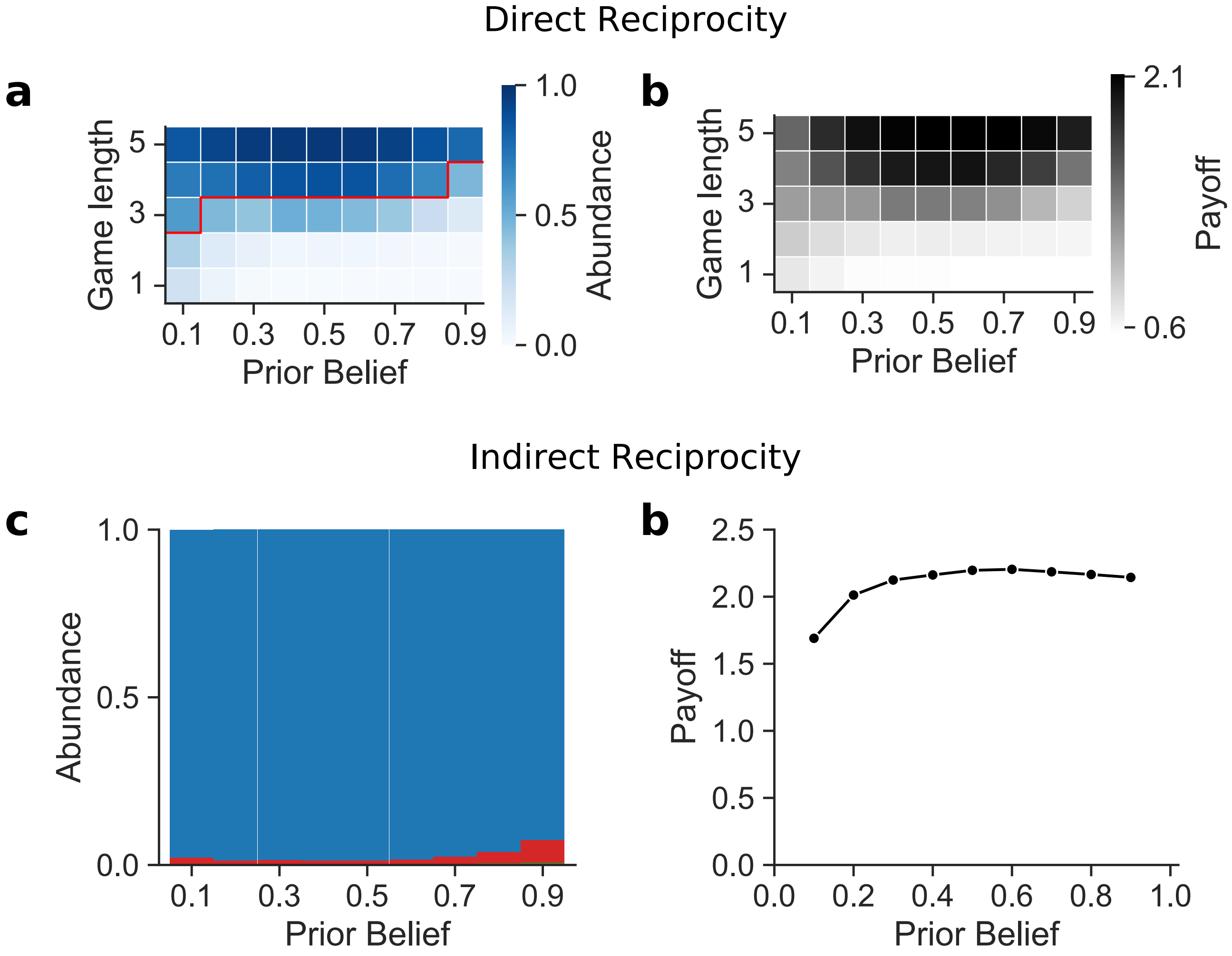

Fig. S1. The evolutionary success of the *Bayesian Reciprocator* is robust to the choice of prior. For priors in the interval of $[0.1, 0.9]$, the *Bayesian Reciprocator* outcompetes *Selfish* and *Altruistic* players in both the repeated but no observability (direct reciprocity, a,b) and one-shot but observable (indirect reciprocity, c,d) *Game Generator*. In the repeated *Game Generator*, the *Bayesian Reciprocator* emerges after a game length of between three and five repetitions (a) and increases the average payoff to each player (b). In the one-shot *Game Generator* with full observability, the *Bayesian Reciprocator* is the most abundant strategy (c) and yields populations with high levels of cooperation (d) for all priors in the interval $[0.1, 0.9]$.

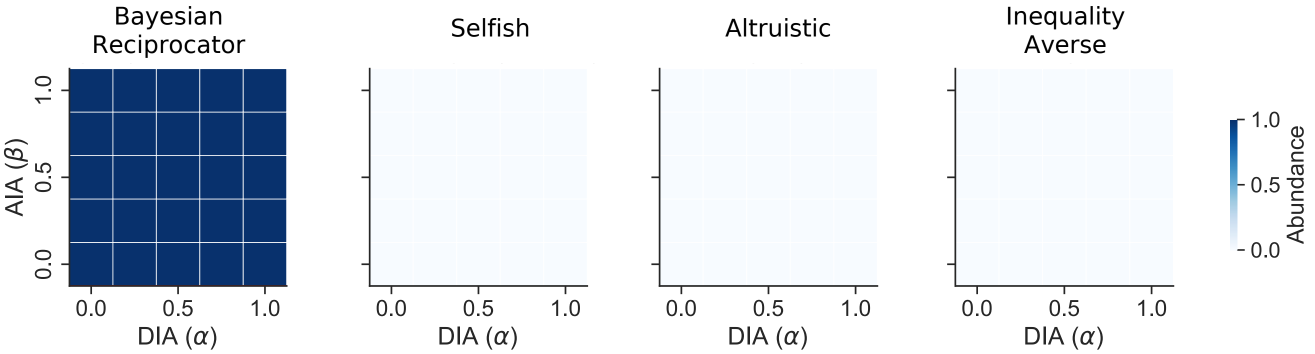

Fig. S2. The *Bayesian Reciprocator* outcompetes *Inequality Averse* players in the repeated *Game Generator*. Let $\mathcal{R}_i$ be the cumulative sum of rewards that player i will have received after the current decision is made. The *Inequality Averse* player then acts according to the following utility function: $U_i = \mathcal{R}_i + \sum_{j \neq i} \mathcal{R}_j - \frac{\alpha}{n-1} \sum_{j \neq i} \max(\mathcal{R}_i - \mathcal{R}_j, 0) - \frac{\beta}{n-1} \sum_{j \neq i} \max(\mathcal{R}_j - \mathcal{R}_i, 0)$ which follows the form of Fehr & Schmidt's model of inequality aversion but replaces the single stage payoffs with the cumulative joint reward (1). At least in principle, this could lead to conditional cooperation under the right parameterization: if a partner is exploiting the Inequality Averse player, disadvantageous inequality aversion (DIA, an aversion to the other partner receiving comparatively more, controlled by α) should prevent the player from continuing to cooperate without reciprocity. Advantageous inequality aversion (AIA, an aversion to receiving more than the other player, controlled by β) should prevent the player from acting overly selfish and enable cooperation to get off the ground. Taken together, these two additional terms could incentivize the player to keep the total reward roughly balanced, low when their partner is not cooperative (e.g., when paired with the *Selfish* player) and high when their partner is cooperative (e.g., when paired with the *Altruistic* player). Across all 25 different parameterizations of the *Inequality Averse* player (five settings of DIA and five settings of AIA), it never outcompetes the *Bayesian Reciprocator*. Simulations were done in the *Game Generator* with a game length of 9 and a probability of action error of 0.025. Heatmaps show the relative abundance of each of the four players at steady state.

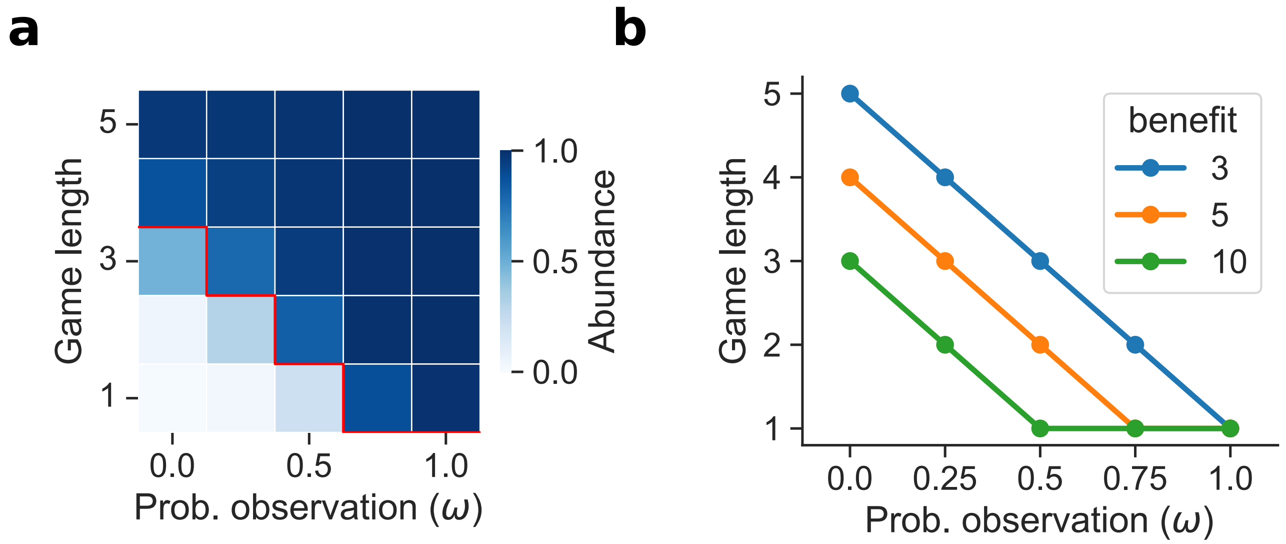

Fig. S3. Observation and interaction mutually support cooperation. (a) Heatmap shows the proportion of *Bayesian Reciprocator* in equilibrium for different game lengths and probability of observations. (b) The minimal number of interactions needed for *Bayesian Reciprocator* to be the modal player at steady state. Different lines show different benefit parameters in the *Game Generator*, while the cost parameter was always fixed at 1. A higher b/c ratio requires fewer repeated interactions for every level of observation.

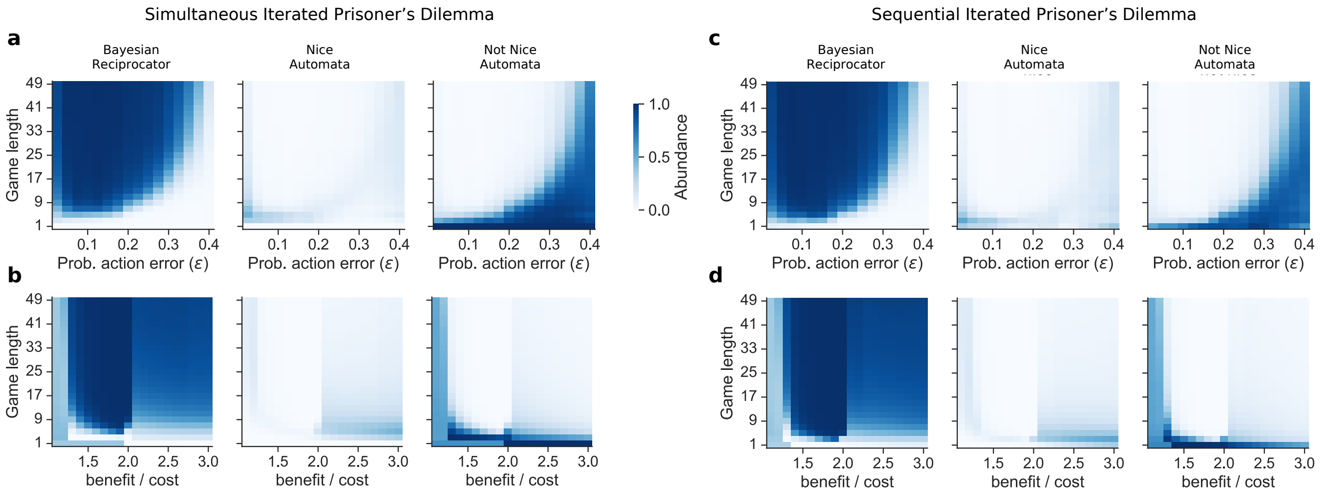

Fig. S4. The Bayesian Reciprocator outcompetes the "nice" automata strategies shown in Figure 6 in the simultaneous (a,b) and sequential (c,d) iterated prisoners dilemma (PD) across a wide variety of action errors (a,c) and benefit / cost ratios (b,d). To more clearly show the relative frequency of each type of player at steady state we categorize automata into Nice and Not Nice (2). Nice automata are those that cooperate on the first move ($p_0 = 1$) and cooperate again if both players cooperated ($p_c c = 1$), Not Nice automata are all others. Heatmaps show the abundance of *Bayesian Reciprocator*, Nice Automata (AllC, TFT, GTFT, WSLS, Forgiver), and Not Nice Automata (AllD, Extortion). When the game length is short, the probability of action error is high, or the benefit / cost is low, Not Nice automata outcompete other players. For longer game lengths and across a wide range of action errors and cost / benefit ratios, the *Bayesian Reciprocator* was the most abundant.

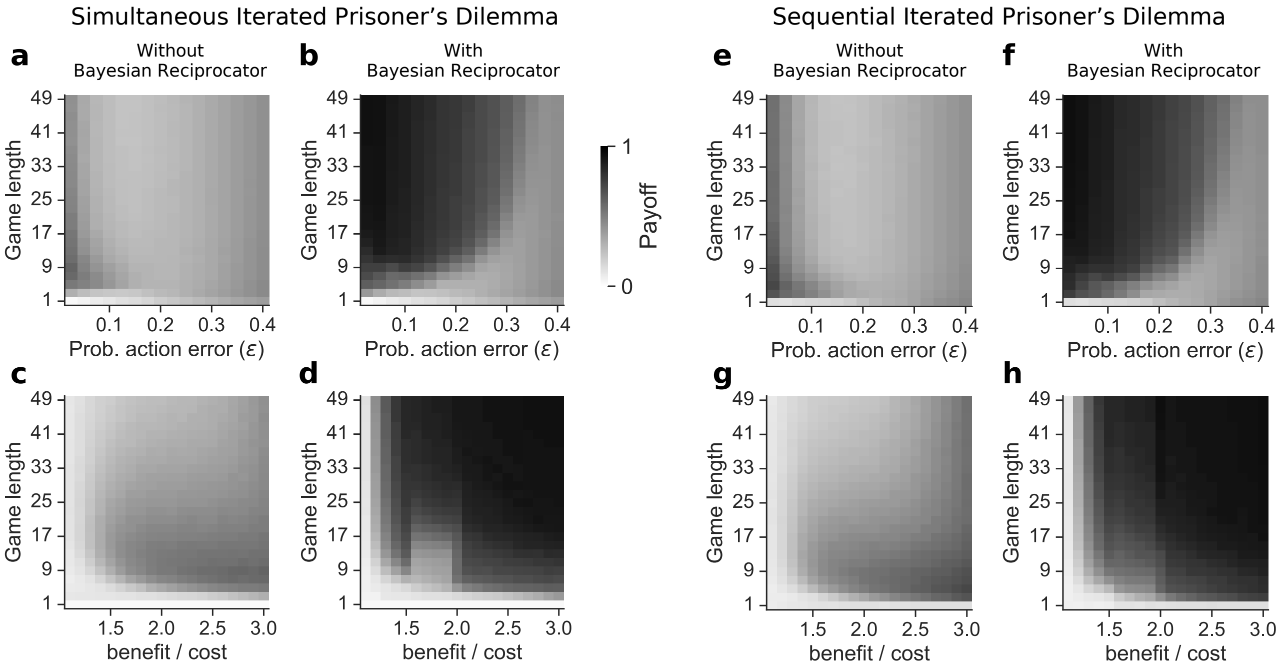

Fig. S5. The *Bayesian Reciprocator* widens the range of parameters that enable cooperation in the simultaneous (a-d) and sequential (e-h) IPD. The population of players is all memory-1 automata (3). Heatmaps show the average payoffs to players in the population at steady state (normalized between 0 and 1). Without the *Bayesian Reciprocator*, cooperation (darker grey regions correspond to higher population payoffs) is limited to long game lengths, low probabilities of action errors, and high benefit / cost ratios (a,c,e,g). With the *Bayesian Reciprocator*, cooperation emerges across a wider range of environmental parameters, and the cooperation that does occur is closer to the maximum, i.e., the grey regions are darker (b,d,f,h).

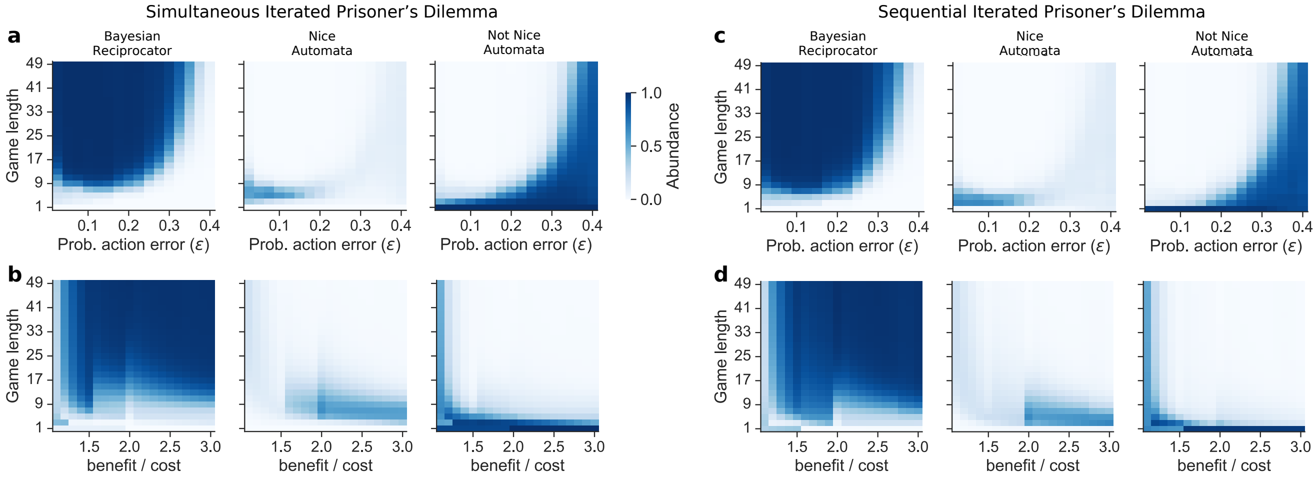

Fig. S6. The Bayesian Reciprocator outcompetes deterministic memory-1 automata strategies (3) in the simultaneous (a,b) and sequential (c,d) iterated prisoners dilemma (PD) across a wide variety of action errors (a,c) and benefit / cost ratios (b,d). Of the 26 deterministic memory-1 automata strategies, 5/26 are Nice, and 21/26 are Not Nice. There is a small region where game lengths are short, errors are low, and benefit/cost is high that we see a mixture of nice automata and the *Bayesian Reciprocator*. See Figure S4 for additional details.

References

1. E Fehr, KM Schmidt, A theory of fairness, competition, and cooperation. *The quarterly journal economics* **114**, 817–868 (1999).
2. C Hilbe, A Traulsen, K Sigmund, Partners or rivals? strategies for the iterated prisoner's dilemma. *Games economic behavior* **92**, 41–52 (2015).
3. BM Zagorsky, JG Reiter, K Chatterjee, MA Nowak, Forgiver triumphs in alternating prisoner's dilemma. *PloS one* **8**, e80814 (2013).
